# A three-tier AI solution for equitable glaucoma diagnosis across China’s hierarchical healthcare system

**DOI:** 10.1038/s41746-025-01835-4

**Published:** 2025-07-03

**Authors:** Yi Zhou, Haitao Nie, Xinyu Gong, Minhui Dai, Zhaohong Guo, Xiaoling Deng, Mengyang Li, Yong Liu, Lingyu Sun, Xiangyi Tang, Ling Zhou, Zhiyao Tang, Ziqing Xia, Lemeng Feng, Wulong Zhang, Qingqing Yi, Xiaobo Xia, Bin Xie, Weitao Song

**Affiliations:** 1 Eye Center of Xiangya Hospital, Central South University, Changsha, Hunan China; 2 Hunan Key Laboratory of Ophthalmology, Central South University, Changsha, Hunan China; 3 National Clinical Research Center for Geriatric Disorders, Xiangya Hospital, Central South University, Changsha, Hunan China; 4 School of Automation, Central South University, Changsha, Hunan China; 5 Department of Ophthalmology, Yiyang Central Hospital, Yiyang, Hunan China; 6 Department of Ophthalmology, Taojiang County People’s Hospital, Taojiang, Hunan China

**Keywords:** Optic nerve diseases, Eye manifestations

## Abstract

Artificial intelligence (AI) offers a solution to glaucoma care inequities driven by uneven resource distribution, but its real-world implementation remains limited. Here, we introduce Multi-Glau, an three-tier AI system tailored to China’s hierarchical healthcare system to promote health equity in glaucoma care, even in settings with limited equipment. The system comprises three modules: (1) a screening module for primary hospitals that eliminates reliance on imaging; (2) a pre-diagnosis module for handling incomplete data in secondary hospitals, and (3) a definitive diagnosis module for the precise diagnosis of glaucoma severity in tertiary hospitals. Multi-Glau achieved high performance (AUC: 0.9254 for screening, 0.8650 for pre-diagnosis, and 0.9516 for definitive diagnosis), with its generalizability confirmed through multicenter validation. Multi-Glau outperformed state-of-the-art models, particularly in handling missing data and providing precise glaucoma severity diagnosis, while improving ophthalmologists’ performance. These results demonstrate Multi-Glau’s potential to bridge diagnostic gaps across hospital tiers and enhance equitable healthcare access.

## Introduction

In China, equitable healthcare delivery faces challenges due to the nation’s vast population and the uneven distribution of medical resources^1,2^—particularly in specialized, expertise-intensive fields such as glaucoma care^3–6^. This imbalance is especially pronounced between urban and underserved populations. To bridge this gap, the Chinese government established a three-tiered healthcare system in 2015, systematically interlinking resources of primary, secondary, and tertiary hospitals with their designated functions^7–10^: suspected glaucoma patients are screened in local rural/urban primary facilities, referred to secondary hospitals in developed regions, and ultimately receive definitive care at tertiary hospitals^11^. However, there is still a certain gap in the implementation of this policy in the field of glaucoma.

Addressing glaucoma care equity through artificial intelligence (AI) is an increasingly critical need. However, AI development has also revealed significant challenges^12–16^, as highlighted by recent concerns about “not teaching AI health disparities” in ophthalmology guidelines^17^. Current AI-driven glaucoma models lack a multi-tiered framework integrated with public health strategies. Most existing models focus on diagnostic fairness within a single hospital tier, primarily supporting screening tasks (glaucomatous vs. healthy eyes) in primary care settings^18–20^. Yet, they fail to address the distinct demands across different healthcare levels. While tertiary hospitals in urban areas require precise visual field severity staging for treatment planning^11^, secondary hospitals in rural regions prioritize referral triage^7^. This fragmentation underscores the need for a systemic AI framework that can coordinate resources across all healthcare tiers rather than functioning in isolated silos, ultimately promoting equitable glaucoma care delivery.

Another major limitation lies in data availability. Current AI models are predominantly developed using “complete” and high-quality multimodal data from well-equipped tertiary hospitals, which may limit their ability to capture real-world complexities, leading to bias and exacerbating disparities^19,21^. In contrast, secondary and primary hospitals often lack essential diagnostic equipment (e.g., visual field tests and optical coherence tomography) and trained ophthalmologists, making accurate diagnosis challenging^7,11^. To promote equitable glaucoma care, AI models must be designed to accommodate the fragmented data realities of these settings, where missing data are common^1,22^.

In this study, we propose Multi-Glau, a multifunctional system designed based on China’s three-tiered healthcare policy to promote equitable development in glaucoma care. It achieves three key objectives: (1) performing screening in primary hospitals using low-cost and easily obtainable clinical features, (2) enabling pre-diagnosis in secondary hospitals based on incomplete data, and (3) providing accurate four-stage glaucoma classification for definitive diagnosis in tertiary hospitals. Its strong performance demonstrates the potential to deliver equitable glaucoma care across all hospital tiers in China and in other regions facing similar healthcare disparities.

## Results

### Overview of study design, data collection and ground truth labeling

**Fig. 1:**
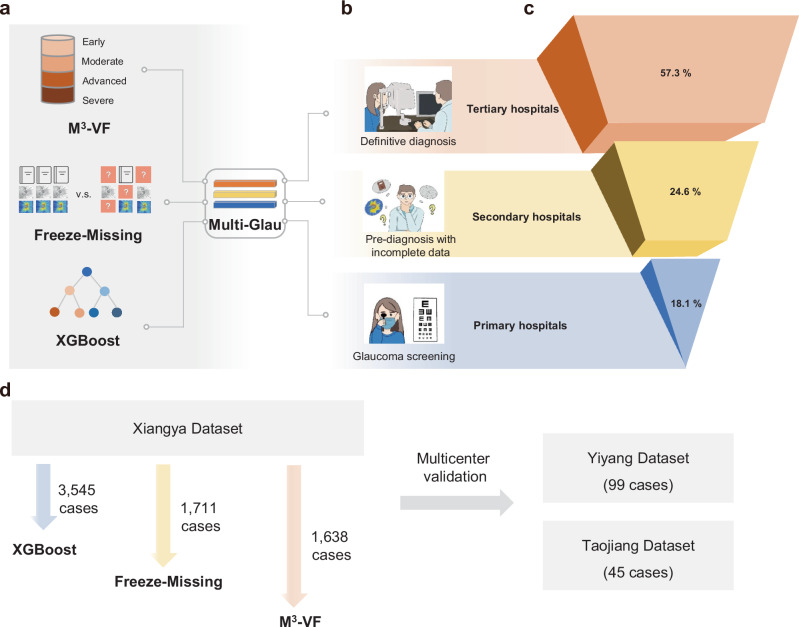
Diagram of the Multi-Glau System for the three-tiered healthcare system in China. The design and goals of Multi-Glau, along with the datasets used for system development and multicenter validation. **a** Components of the Multi-Glau system. **b** Workflow of the Multi-Glau system, highlighting its designated functions across the three-tiered glaucoma diagnosis model. **c** Distribution of medical resources, illustrating the concentration of resources in tertiary hospitals and the relative scarcity in secondary and primary hospitals. The ratios representing medical resources are adapted from Yip et al.^7^ **d** Data collection for system development and multicenter validation.

The Multi-Glau integrates three modules to address designated functions of glaucoma diagnosis at three tiers of hospitals: screening, pre-diagnosis, and definitive diagnosis (Fig. 1a–d). The screening function utilizes the XGBoost classifier module to screen for glaucoma in primary hospitals, using epidemiological data (such as age and gender) and ophthalmic parameters such as intraocular pressure (IOP), best-corrected visual acuity (BCVA), and cup-to-disk ratio (CDR). We chose these five numerical data points instead of fundus photographs mainly considering the convenience of collecting such information among elderly patients in remote rural areas of China. For example, community doctors conducting telemedicine consultations and telephone follow-ups cannot exchange images. The pre-diagnosis function is achieved through the Freeze-Missing module, which handles incomplete data in secondary hospitals and categorizes visual field damage into early and advanced categories. The definitive diagnosis function employs through the M^3^-VF module, which incorporates a multi-perspective, multi-modal, and multi-stage attention mechanism in tertiary hospitals, grading visual field damage into early, moderate, advanced, and severe categories using epidemiological data, ophthalmic parameters, fundus photographs, and optical coherence tomography (OCT) scans.

We collected data from the electronic medical records (EMRs) of 27,255 glaucoma patients at the Ophthalmology Center of Xiangya Hospital Central South University from 2017 to 2024. After removing duplicates and retaining the most recent visit record, the dataset was reduced to 8358 unique cases. To minimize the potential influence of confounding factors on glaucoma diagnosis, VF grading, and IOP, we excluded patients with co-existing ocular diseases or secondary glaucomas, such as high myopia, diabetic retinopathy, hypertensive retinopathy, uveitis, traumatic glaucoma, and neovascular glaucoma. For examples, high myopia induces anatomical changes in the optic nerve head that can obscure glaucoma-specific features, such as neuroretinal rim thinning and RNFL changes. By excluding these cases, we avoided confounding structural alterations and maintained the clarity and reliability of our grading system. Additionally, we excluded low-quality OCT and visual field results to ensure data integrity (full inclusion and exclusion criteria are detailed in the “Methods” section).

The final paired dataset consisted of 3545 cases, and the participants underwent comprehensive assessments as shown in Supplementary Fig. 1. The dataset was classified into four categories of visual field damage: early (1198), moderate (655), advanced (535), and severe (1157) VF damage, collected from Xiangya Hospital Central South University. For multicenter validation, 99 cases were collected from Yiyang Central Hospital and 45 cases from Taojiang County People’s Hospital (Fig. 1d).

Ground truth labeling for the three modules was based on a standardized process illustrated in Supplementary Fig. 1. In the screening task, glaucoma cases were annotated by reviewing EMRs with ICD-10 codes beginning with H40, and these annotations were verified by glaucoma specialists. The severity of visual field damage was categorized into four groups based on the mean deviation (MD) value: early (MD ≥ −6 dB), moderate (−6 to −12 dB), advanced (−12 to −20 dB), and severe (MD ≤ −20 dB), adapting the Hodapp-Anderson-Parrish glaucoma staging system^23,24^. For the pre-diagnosis task, glaucoma severity was broadly classified into early and serious categories, with “serious” encompassing moderate, advanced, and severe stages. In the definitive diagnosis task, glaucoma severity was finely graded into early, moderate, advanced, and severe categories.

### Data distribution

**Fig. 2:**
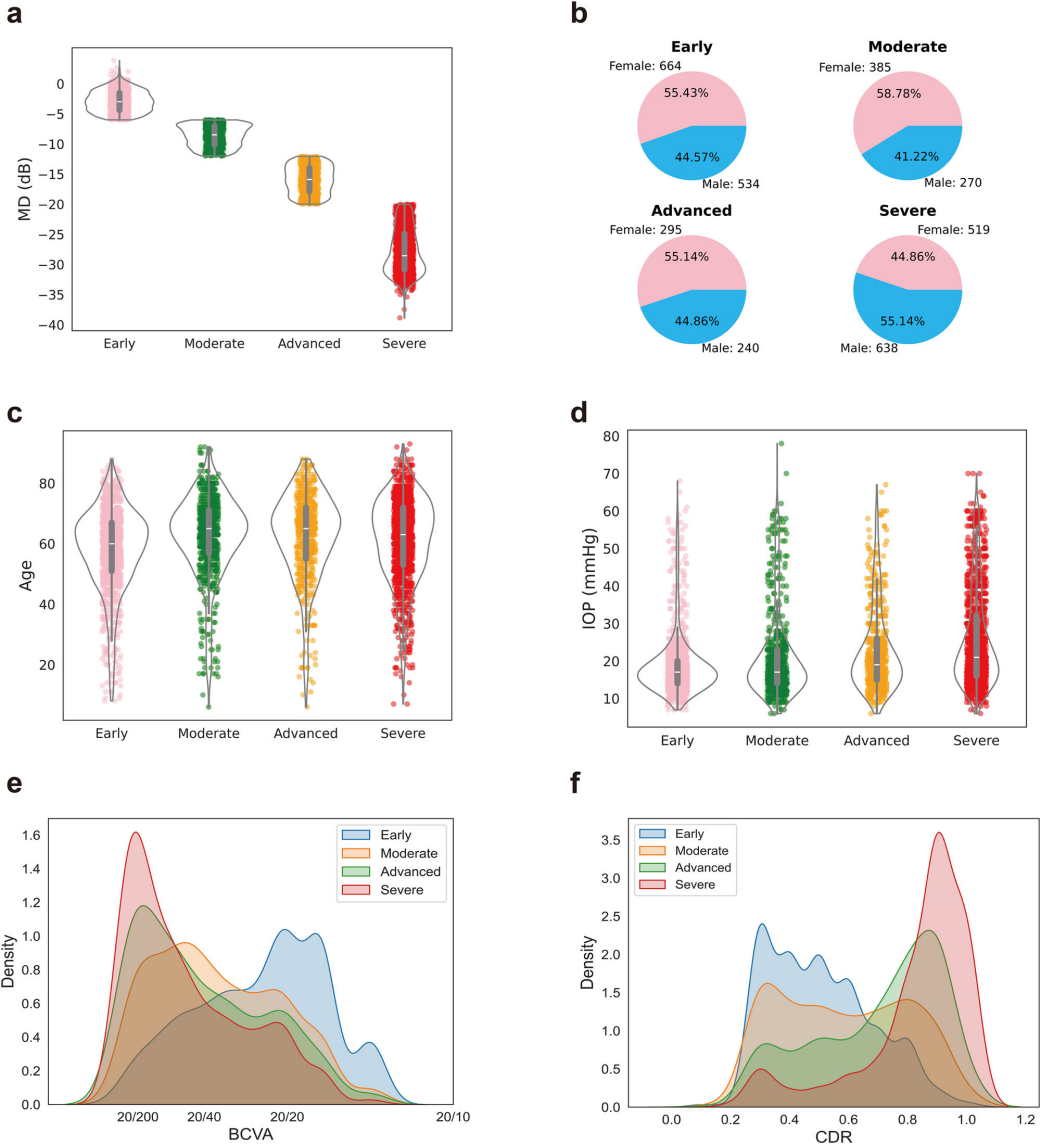
Description of the glaucomatous visual field damage severity grading datasets. Violin plots, pie charts, and kernel density estimation (KDE) plots for exploratory data analysis using data grouped into four categories: early, moderate, advanced, and severe. Distribution of **a** median deviation values, **b** gender, **c** age, **d** intraocular pressure, **e** best-corrected visual acuity and **f** cup-to-disc ratio.

The mean MD values of early, moderate, advanced, and severe visual field damage groups were −2.91 dB, −8.64 dB, −15.91 dB and −27.77 dB (Fig. 2a). The gender distribution in the severe VFD group exhibited a marginal difference from the remaining 3 categories, featuring a diminished proportion of female patients (~44.86%, Fig. 2b). The mean age from early to severe visual field severity groups were 58.0, 62.5, 62.4 and 61.4 years old, and the mean IOP were 18.64 mmHg, 20.88 mmHg, 22.71 mmHg, 25.79 mmHg, respectively (Fig. 2c, d). The density graph indicated that patients with advanced to severe visual field damage were more prone to exhibit poorer visual acuity (Fig. 2e) and higher CDR (Fig. 2f). The descriptions of data collected from Xiangya (Supplementary Tables 1–3), Taojiang (Supplementary Tables 4–6) and Yiyang (Supplementary Tables 7–9) are presented.

### Performance of the screening modules

**Fig. 3:**
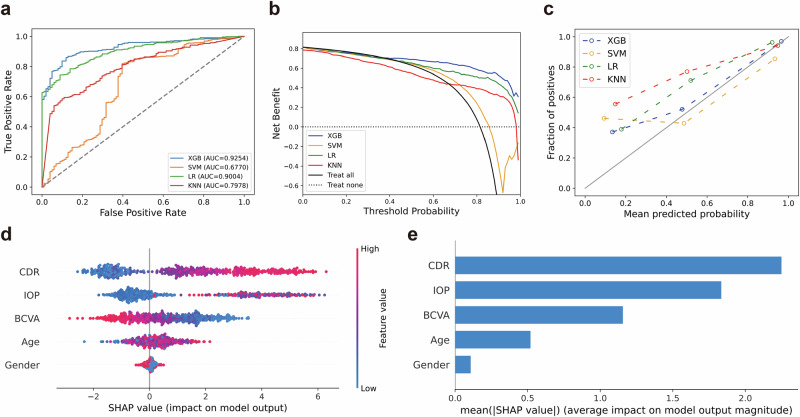
Performance of the XGB screening module in primary hospitals. Comparison of the XGB screening module with benchmark models and interpretability analysis of XGB module on screening glaucoma. **a** The AUC curve, **b** clinical benefit curve, and **c** calibration curve for the XGB, SVM, KNN, and LR classifiers. **d** The SHAP explainer interpreting feature importance for the XGB screening module. **e** The mean SHAP values and ranking of each clinical feature in the XGB screening module.

We conducted a comparison of screening performance across four models: K-Nearest Neighbors Classifier (KNN)^25^, Support Vector Machine (SVM)^26^^,^ Logistic Regression (LR)^27^, and XGBoost (XGB)^28^. The results in Table 1 indicate that XGB exhibited the best predictive performance (sensitivity = 0.8857, AUC = 0.9254). Conversely, SVM demonstrated the poorest predictive performance (specificity = 0.2871). Supplementary Table 10 presents the cross-validation results. The ROC curve reflects a model’s classification ability at different thresholds. Figure 3a shows that XGB achieves a higher true positive rate (TPR) than other models at the same false positive rate (FPR), indicating better detection of positive samples without increasing false positives. In Fig. 3b, the XGB curve is the outermost and consistently above the “Treat none” line, signifying higher net benefits at any threshold, with all benefits being positive. Both XGB and other models underestimate risk in the low probability range (Fig. 3c), but XGB closely matches the actual risk in the high probability range. Figure 3d, e represent the SHapley Additive exPlanations (SHAP) results. SHAP is an approach to explain the output of machine learning models by assigning each feature its contribution to the prediction as a Shapley value^29^. Fig. 3d shows that higher CDR and IOP increase glaucoma likelihood, while lower BCVA is linked to glaucoma. Age and gender have minimal impact on the XGB module to screen glaucoma among healthy individuals. Figure 3e ranks the five features impacting the XGB screening module: CDR > IOP > BCVA > Age > Gender.

**Table 1:** Performance comparison between the XGB screening module and benchmark models

Classifiers	Accuracy	Sensitivity	Specificity	AUC
LR	0.8391	0.8632	0.7327	0.9004
KNN	0.7331	0.7444	0.6832	0.7978
SVM	0.8263	**0.9484**	0.2871	0.6770
XGB	**0.8739**	0.8857	**0.8218**	**0.9254**

The best values are highlighted in bold, and values below 0.6 are underlined.

We assessed the target sensitivity and specificity of the screening module (XGB), along with its multicenter validation. The screening module maintained strong performance across different thresholds (Table 2). At a target sensitivity of 0.8500, the specificity was 0.8812. Notably, with a target specificity of 0.9500, the sensitivity was 0.7220. All metrics for XGB in the two multicenter validation sets exceeded 0.8, and the AUC values were greater than 0.9 (Table 3). It is noteworthy that the sensitivity of XGB on the Taojiang dataset is 0.9750, and the specificity on the Yiyang dataset is 1.0000.

**Table 2:** Performance of the XGB screening module under target sensitivity and specificity

Target Sensitivity	Specificity	Accuracy	Target Specificity	Sensitivity	Accuracy
0.8000	0.9010	0.8483	0.8000	0.8901	0.8757
0.8500	0.8812	0.8629	0.8500	0.8677	0.8647
0.9000	0.7723	0.8775	0.9000	0.7982	0.8172
0.9500	0.6040	0.8867	0.9500	0.7220	0.7642

**Table 3:** Multicenter validation of the XGB screening module

Datasets	Accuracy	Sensitivity	Specificity	AUC
Taojiang	0.9556	0.9750	0.8000	0.9550
Yiyang	0.8788	0.8636	1.0000	0.9318

### Performance of the pre-diagnosis module

**Fig. 4:**
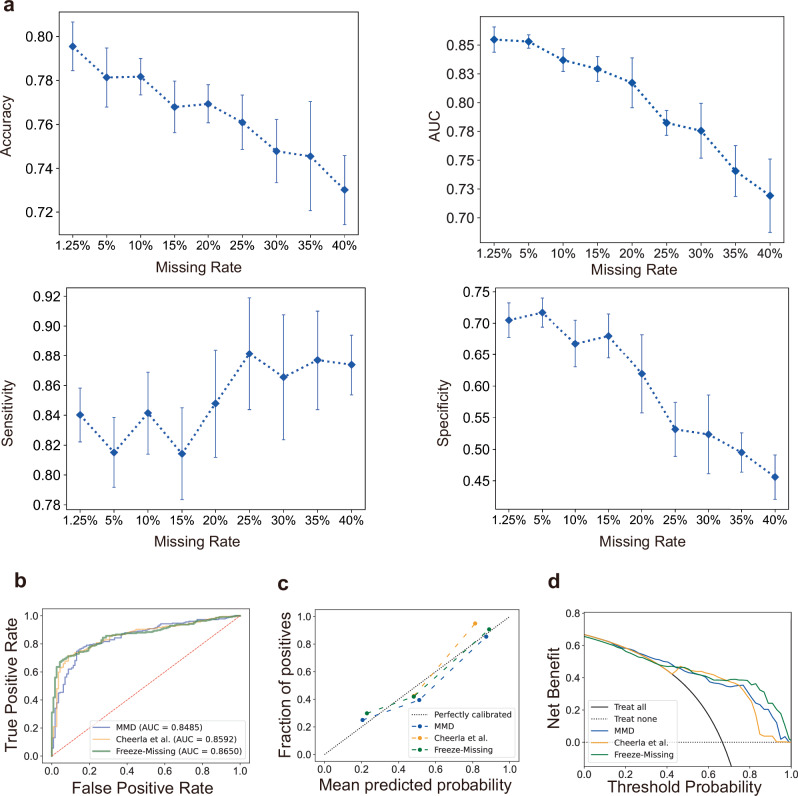
Performance the Freeze-Missing module in secondary hospitals. Performance of the Freeze-Missing module on multiple missing datasets and its performance comparison with SOTA models. **a** The line graphs showing accuracy, sensitivity, specificity, and AUC for the Freeze-Missing module, including mean and standard deviation at various missing rates. **b** The AUC curve, **c** calibration curve, and **d** clinical benefit curve for the Freeze-Missing module versus SOTA models (MMD model, Cheerla et al.’s model).

We evaluated the performance of the Freeze-Missing module under varying missing rates. A major challenge faced by secondary hospitals is the unavailability of imaging data due to limited diagnostic equipment, leaving only a few basic clinical parameters (e.g., IOP, BCVA) accessible. To simulate this scenario, we introduced a completely random image missingness mechanism, constructing datasets with missingness rates ranging from 5% to 40% in 5% increments and generating 10 independent datasets for each level. As shown in Fig. 4a and Table 4, as the missing data rate increases, accuracy, specificity, and AUC exhibit a fluctuating decline, while sensitivity shows a fluctuating increase. The changes in module performance primarily stem from data distribution variations caused by completely random missing values. Pre-diagnosis requires high sensitivity. The Freeze-Missing module achieves a sensitivity of 0.8738 at a data missing rate of up to 40%. Supplementary Fig. 2 presents the AUC curves at different missing rates, while Supplementary Fig. 3 displays the confusion matrices.

**Table 4:** Performance testing of the Freeze-Missing module

Missing rates	Accuracy	Sensitivity	Specificity	AUC
1.25%	0.7978 ± 0.0046	0.8419 ± 0.0146	0.7085 ± 0.0268	0.8551 ± 0.0110
5%	0.7813 ± 0.0141	0.8151 ± 0.0246	0.7169 ± 0.0244	0.8530 ± 0.0061
10%	0.7833 ± 0.0091	0.8470 ± 0.0295	0.6617 ± 0.0516	0.8348 ± 0.0085
15%	0.7679 ± 0.0124	0.8142 ± 0.0324	0.6797 ± 0.0370	0.8292 ± 0.0114
20%	0.7694 ± 0.0092	0.8479 ± 0.0383	0.6196 ± 0.0660	0.8172 ± 0.0229
25%	0.7609 ± 0.0130	0.8813 ± 0.0396	0.5314 ± 0.0450	0.7824 ± 0.0114
30%	0.7478 ± 0.0167	0.8656 ± 0.0485	0.5233 ± 0.0720	0.7756 ± 0.0275
35%	0.7455 ± 0.0263	0.8769 ± 0.0349	0.4949 ± 0.0327	0.7406 ± 0.0233
40%	0.7300 ± 0.0166	0.8738 ± 0.0212	0.4559 ± 0.0370	0.7192 ± 0.0336

The table reports the mean and standard deviation, and values below 0.6 are underlined.

Ablation experiments on Se-Conv, mean fusion, and concatenation fusion were conducted to evaluate design choices within the Freeze-Missing module (Table 5). Mean fusion and concatenation fusion methods exhibit similar performance levels. The Se-Conv + Mean method did not improve the model’s performance; instead, there was a slight performance drop compared to using only the mean fusion. The performance decline arises from the mean fusion, which computes the global average of feature vectors, while Se-Conv further filters information at the channel level. These processes result in excessive information blurring. On the contrary, Se-Conv + Concatenation outperformed Se-Conv + Mean in all metrics, especially with specificity exceeding by 3.33%. This is because the effective features extracted by Se-Conv are maximally preserved through concatenation information fusion.

**Table 5:** Ablation study of Freeze-Missing module

Ablation Module	Accuracy	Sensitivity	Specificity	AUC
Mean	0.7879 ± 0.0139	0.8410 ± 0.0259	0.6804 ± 0.0668	0.8507 ± 0.0109
	*0.7697*	*0.8690*	*0.5702*	*0.8527*
Concatenation	0.7937 ± 0.0160	0.8349 ± 0.0219	0.7102 ± 0.0236	0.8515 ± 0.0096
	*0.7953*	*0.8428*	*0.6991*	*0.8449*
Se-Conv + Mean	0.7843 ± 0.0177	0.8384 ± 0.0459	0.6752 ± 0.0789	0.8522 ± 0.0121
	*0.7924*	*0.8341*	*0.7079*	*0.8561*
Se-Conv + Concatenation	0.7978 ± 0.0046	0.8419 ± 0.0146	0.7085 ± 0.0268	0.8551 ± 0.0110
	*0.8017*	*0.8428*	*0.7193*	*0.8650*

The upright numbers represent the means and standard deviations of the five-fold cross-validation results. Italicized numbers represent the results of the best model during cross-validation. Values below 0.6 are underlined.

We compared the performance of the Freeze-Missing module with SOTA models. Cui et al.’s MMD model^30^ minimizes reconstruction loss to enhance representation learning despite missing data. Cheerla et al.’s model^31^ projects multimodal information into a unified feature space and compensates for information loss by weighting the complete modalities. The results (Table 6) of the 5-fold cross-validation show that Freeze-Missing achieved the best predictive performance (sensitivity = 0.8419, AUC = 0.8551), followed by Cheerla et al.’s model (sensitivity = 0.8271, AUC = 0.8441), while MMD performed the worst. MMD’s ability to identify negative samples is limited (specificity = 0.4956). Using accuracy to select the best model from five-fold cross-validation, the performance ranking is as follows: Freeze-Missing > Cheerla’s model > MMD. There are significant differences in specificity among the three models (Freeze-Missing: 0.7193, Cheerla’s model: 0.6106, MMD: 0.5487). The ROC curve (Fig. 4b) shows that Freeze-Missing has a higher true positive rate than the SOTA models in the low false positive rate range, while all models perform similarly in other ranges. The calibration curve (Fig. 4c) indicates that Cheerla’s model has the lowest calibration, while the curves for Freeze-Missing and MMD are closer to the reference line. The clinical benefits of the three models show little difference in the low probability range (Fig. 4d), with Freeze-Missing offering greater benefits in the high probability range.

**Table 6:** Performance comparisons between Freeze-Missing and SOTA models

Methods	Accuracy	Sensitivity	Specificity	AUC
MMD	0.7754 ± 0.0200	**0.9135** ± **0.0189**	0.4956 ± 0.0468	0.8366 ± 0.0178
	*0.8012*	*0.9258*	*0.5487*	*0.8485*
*Cheerla et al*.	0.7908 ± 0.0134	0.8271 ± 0.0539	**0.7172** ± **0.0710**	0.8441 ± 0.0132
	*0.8128*	*0.9126*	*0.6106*	*0.8592*
Freeze-Missing	**0.7978** ± **0.0046**	0.8419 ± 0.0146	0.7085 ± 0.0268	**0.8551** ± **0.0110**
	*0.8017*	*0.8428*	*0.7193*	*0.8650*

The upright numbers represent the means and standard deviations of the five-fold cross-validation results, with bolded values indicating the best performance. Italicized numbers represent the results of the best model during cross-validation. Values below 0.6 are underlined.

We evaluated the Freeze-Missing module under target sensitivity and specificity settings, as well as in multicenter validation. Table 7 shows that when both sensitivity and specificity are set to 0.8500, all metrics exceed 0.7000. When the target specificity is set to 0.9500, the accuracy still reaches 0.7464. Freeze-Missing obtained an AUC (Table 8) of 0.7438 for the Taojiang dataset and 0.8112 for the Yiyang dataset.

**Table 7:** Performance of the Freeze-Missing module under target sensitivity and specificity

Target Sensitivity	Specificity	Accuracy	Target Specificity	Sensitivity	Accuracy
0.8000	0.7627	0.7872	0.8000	0.7511	0.7697
0.8500	0.7119	0.8076	0.8500	0.7289	0.7726
0.9000	0.4237	0.7376	0.9000	0.7022	0.7755
0.9500	0.2458	0.7085	0.9500	0.6356	0.7464

**Table 8:** Multicenter validation of Freeze-Missing module

Datasets	Accuracy	Sensitivity	Specificity	AUC
Taojiang	0.7111	0.6944	0.7778	0.7438
Yiyang	0.7071	0.7091	0.7045	0.8112

### Performance of the definitive diagnosis module

To investigate the performance gap between single-modal and multimodal inputs, we compared single-stream models with the multimodal M^3^-VF model. Global flow, focused flow, and RNFL flow use data from fundus images derived from OCT scans, fundus images transformed by polar coordinates, and RNFL thickness maps, respectively. Compared to single-stream models, M^3^-VF improves all metrics, with accuracy increasing by at least 20.21%, sensitivity by 20.96%, specificity by 7.28%, and AUC by 15.8% (Table 9). The sensitivity of all three single-stream models was below 0.60, implying a high rate of false negatives, which could lead to delayed detection of glaucomatous visual field damage. The results indicate that using unimodal information alone makes fine-grained four-class classification difficult.

**Table 9:** Comparison of the performance between the single-stream models and M^3^-VF

	Accuracy	Sensitivity	Specificity	AUC
Global Flow	0. 4406 ± 0.0223	0.4097 ± 0.0202	0.8059 ± 0.0083	0.6631 ± 0.0194
Focused Flow	0.4257 ± 0.0243	0.3857 ± 0.0199	0.7992 ± 0.0086	0.6484 ± 0.0093
RNFL Flow	0.5649 ± 0.0250	0.5419 ± 0.0269	0.8492 ± 0.0097	0.7703 ± 0.0184
M^3^-VF	**0.7670** ± **0.0372**	**0.7515** ± **0.0450**	**0.9220** ± **0.0126**	**0.9283** ± **0.0144**

The best value for each metric was highlighted in bold. Values below 0.6 are underlined.

We performed ablation experiments on the M^3^-VF module to evaluate the contributions of CBAM and the transformer encoder (Table 10). The Base model exhibited the weakest performance (accuracy = 0.4742, sensitivity = 0.4455). The Base + CBAM model showed improvements across all metrics compared to the Base model, increasing accuracy, sensitivity, and AUC by more than 11%. This improvement stems from CBAM’s ability to extract higher-quality unimodal information through attention mechanisms at both channel and spatial levels. The Base + transformer encoder model, compared to Base, increased accuracy, sensitivity, and AUC by more than 15%, and compared to Base + CBAM, increased accuracy, sensitivity, and AUC by more than 4%. The role of the transformer encoder is to learn end-to-end fusion weights for multimodal information. Consequently, identifying the optimal fusion method for multimodal data is more critical than merely extracting high-quality unimodal information. The Base + transformer encoder + CBAM (M^3^-VF) model performed the best because it combines the ability to extract high-quality unimodal features and determine the optimal multimodal fusion method simultaneously.

**Table 10:** Ablation study of M^3^-VF

Ablation Module	Accuracy	Sensitivity	Specificity	AUC
Base	0.4742 ± 0.0377	0.4455 ± 0.0345	0.8191 ± 0.0129	0.7136 ± 0.0256
	*0.4907*	*0.4551*	*0.8242*	*0.7432*
Base +CBAM	0.6134 ± 0.0212	0.5883 ± 0.0290	0.8686 ± 0.0079	0.8298 ± 0.0166
	*0.6324*	*0.6202*	*0.8758*	*0.8486*
Base + transformer encoder	0.6694 ± 0.0186	0.6478 ± 0.0276	0.8881 ± 0.0073	0.8723 ± 0.0153
	*0.6947*	*0.6868*	*0.8978*	*0.8899*
Base + transformer encoder+ CBAM (ours)	0.7670 ± 0.0372	0.7515 ± 0.0450	0.9220 ± 0.0126	0.9283 ± 0.0144
	*0.8255*	*0.8210*	*0.9414*	*0.9516*

The upright numbers represent the means and standard deviations of the five-fold cross-validation results. Italicized numbers represent the results of the best model during cross-validation. Values below 0.6 are underlined.

**Fig. 5:**
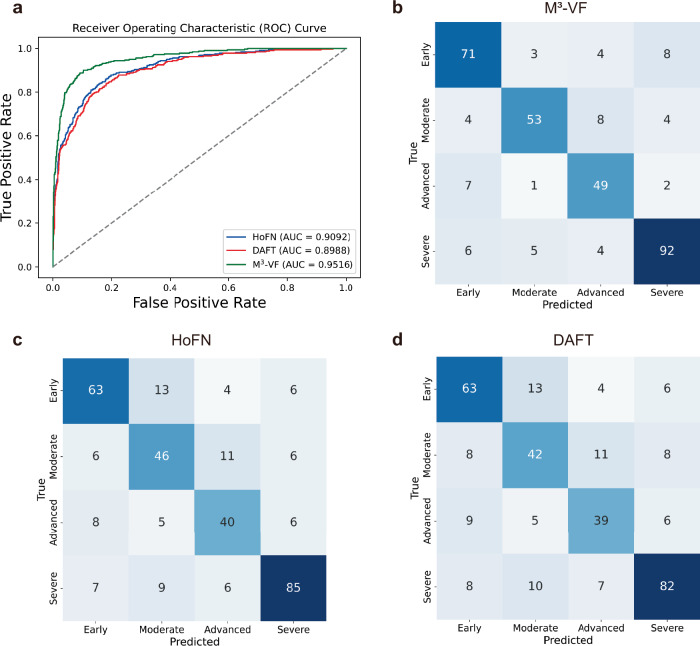
Performance of the M^3^-VF module in tertiary hospitals. Performance comparison between the M^3^-VF module, DAFT model, and HoFN model, with both DAFT and HoFN designed for fine-grained classification. **a** The AUC curve, and the confusion matrixes for **b** M^3^-VF module, **c** HoFN model and **d** DAFT model.

To assess both the classification performance and generalizability of M^3^-VF, we compared it with two state-of-the-art models and conducted multicenter validation (Fig. 5 and Table 11). Dynamic Affine Feature Map Transform (DAFT)^32^ and High-order Factorization Network (HoFN)^33^ were selected as comparison models due to their advanced techniques in multimodal fusion and multi-class classification. Compared to DAFT, M^3^-VF enhanced accuracy by 8%, sensitivity by 9%, specificity by 3%, and AUC by 4% (Table 11). The limited number of numerical features used in this study may not provide DAFT with sufficient information to adjust the feature maps, resulting in suboptimal performance. Compared to HoFN, M^3^-VF enhanced accuracy by 6%, sensitivity by 7%, specificity by 2%, and AUC by 3%. The performance of HoFN is worse, likely due to its reliance solely on concatenation of multimodal features. M^3^-VF maintains a higher true positive rate across nearly all false positive rates (Fig. 5a) and outperforms HoFN and DAFT in correct predictions for each category (Fig. 5b, c, d). Finally, we conducted multicenter validation on M^3^-VF (Table 12). M^3^-VF achieved high AUC values, with 0.9203 on the Taojiang dataset and 0.8982 on the Yiyang dataset.

**Table 11:** Performance comparison between M^3^-VF and the SOTA models

	Accuracy	Sensitivity	Specificity	AUC
DAFT	0.6787 ± 0.0210	0.6556 ± 0.0278	0.8915 ± 0.0080	0.8808 ± 0.0143
	*0.7040*	*0.6922*	*0.9013*	*0.8988*
HoFN	0.7005 ± 0.0225	0.6788 ± 0.0293	0.8988 ± 0.0087	0.8916 ± 0.0138
	*0.7290*	*0.7179*	*0.9098*	*0.9092*
M^3^-VF	**0.7670** ± **0.0372**	**0.7515** ± **0.0450**	**0.9220** ± **0.0126**	**0.9283** ± **0.0144**
	*0.8255*	*0.8210*	*0.9414*	*0.9516*

The upright numbers represent the means and standard deviations of the five-fold cross-validation results, with bolded values indicating the best performance. Italicized numbers represent the results of the best model during cross-validation.

**Table 12:** Multicenter validation of M^3^-VF

Dataset	Accuracy	Sensitivity	Specificity	AUC
Taojiang	0.7333	0.7272	0.9072	0.9203
Yiyang	0.6970	0.7059	0.9044	0.8982

### Deployment, interpretability, and human-computer interaction of Multi-Glau

To enhance clinical decision-making for glaucoma care, the Multi-Glau system has been deployed as both a local tool and a web platform. Multi-Glau supports hospitals at three tiers by addressing distinct needs: screening for general practitioners with basic clinical parameters, pre-diagnosis for secondary hospital doctors managing incomplete data, and definitive diagnosis for specialists in tertiary hospitals.

The Multi-Glau system identifies glaucoma-related structural changes, offering interpretability insights into its decision-making process. An interpretability study combined GradCAM++^34^ to visualize region-level attention with Guided Backprop^35^ to highlight pixel-level attention, and their overlay further enhanced understanding of the system’s decision-making mechanisms. The system identified key structural changes in the optic disc, such as peripapillary hemorrhage, parapapillary hemorrhage, and neuroretinal rim hemorrhage (Supplementary Fig. 4a, 4b, and 4c, respectively). Other glaucoma-related features, including peripapillary atrophy, bayoneting of vessels, and circumlinear baring, were also detected, further supporting Multi-Glau’s contribution to glaucoma care.

**Fig. 6:**
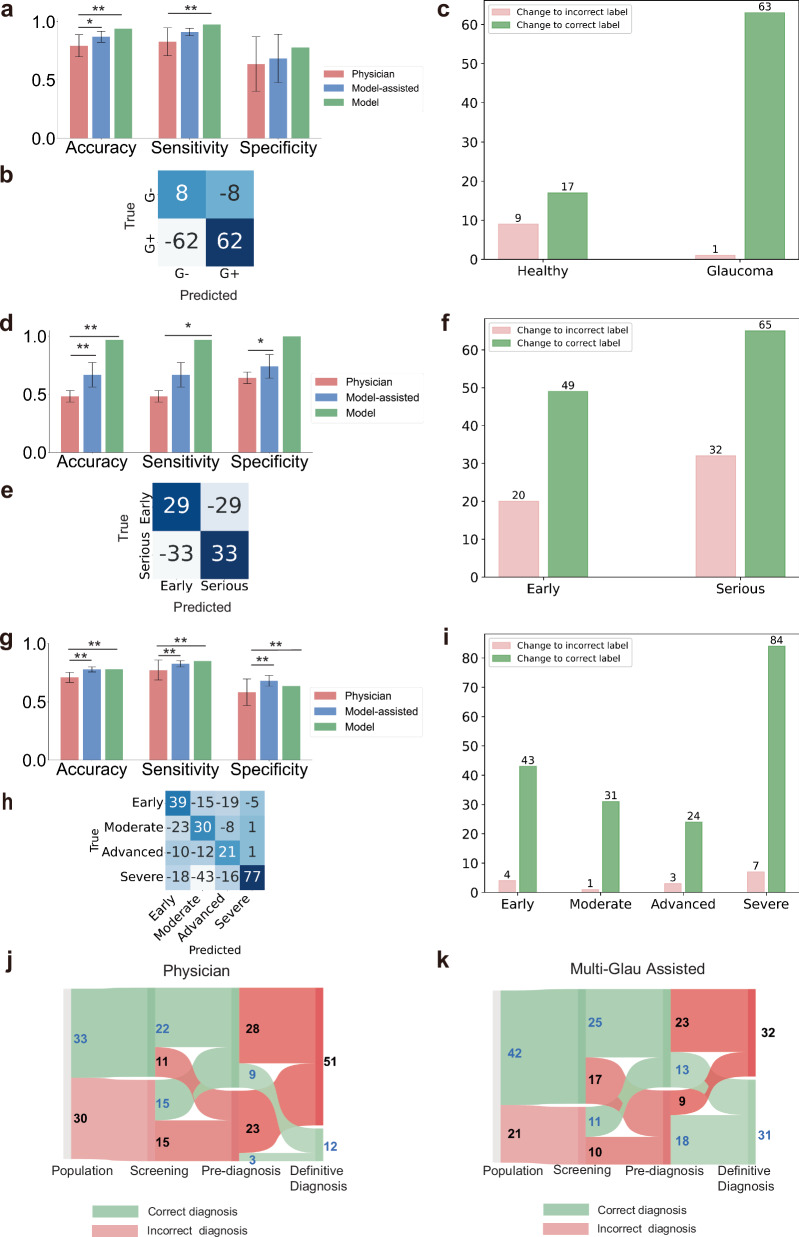
The Multi-Glau system aids physicians in medical decision-making across three tiers of hospitals. Impact of Multi-Glau on physicians’ diagnostic performance across three hospital tiers. Nine physicians (three each from junior, mid-level, and senior levels) independently diagnosed glaucoma cases with and without Multi-Glau assistance. **a–c** Screening task (primary hospitals); **d**–**f** Pre-diagnosis task (secondary hospitals); **g**–**i** Definitive diagnosis task (tertiary hospitals). **a**, **d**, **g** show diagnostic performance; **b**, **e**, **h** show changes in confusion matrices before and after AI assistance; **c**, **f**, **i** show physician decision changes. Sankey diagrams illustrate diagnostic outcomes before (**j**) and after (**k**) Multi-Glau assistance across all three tasks. Green flows indicate correct diagnoses; red flows indicate incorrect diagnoses. **a**–**h** used 100 cases; **j**–**k** used 63 shared cases. *P < 0.05, **P < 0.01, independent samples *t*-test.

The system’s interpretability significantly benefits physicians’ decision-making. To evaluate its effectiveness, nine ophthalmology experts (junior, intermediate, and senior levels) from three-tiered hospitals assessed 100 samples independently. They then performed a second assessment after reviewing Multi-Glau’s interpretability diagrams. The results, summarized in Fig. 6 and Supplementary Tables 11–13, demonstrate that the collaboration between Multi-Glau and physicians significantly outperformed decisions made by physicians alone. Diagnostic accuracy showed the greatest improvement in definitive diagnosis tasks compared to other tasks (*P* < 0.01, independent samples t-test, Fig. 6a, d, g). Additionally, Multi-Glau had a greater impact on decision-making in cases of severe visual field loss than in milder cases (Fig. 6b, e, h). Across all tasks, the number of correct decision changes exceeded incorrect ones, highlighting the positive influence of Multi-Glau’s interpretability (Fig. 6c, f, i).

Simulating the referral process across three-tiered hospitals highlighted Multi-Glau’s potential to transform decision-making. Without Multi-Glau, only 3 of 26 samples incorrectly diagnosed during pre-diagnosis were corrected at the definitive diagnosis stage (Fig. 6j). With Multi-Glau, this number increased to 18 of 27 samples (Fig. 6k), underscoring its ability to enhance diagnostic accuracy and streamline patient management across healthcare tiers.

## Discussion

In China’s three-tiered healthcare system, equity in glaucoma care refers to ensuring that all individuals receive timely and appropriate diagnostic services, regardless of their geographic location or the level of the healthcare facility they attend. To promote equitable implementation, this study addresses disparities in resource availability and service capacity across primary, secondary, and tertiary institutions by proposing a tiered-design AI system. This system integrates three functional modules—XGBoost for screening, Freeze-Missing for pre-diagnosis, and M³-VF for definitive diagnosis—to address challenges posed by incomplete multimodal data and to enable accurate classification of glaucoma severity. Multi-Glau’s performance demonstrated its effectiveness, while human-AI interaction experiments demonstrated significant improvements in detecting severe cases, particularly in resource-limited settings.

To our knowledge, this is the first study to systematically address incomplete multimodal data in glaucoma diagnosis. Existing models exclusively depend on comprehensive datasets (e.g., OCT, VF, CFP)^36–40^, a requirement that confines their utility to well-resourced tertiary hospitals in high-income regions—thereby exacerbating disparities in glaucoma care accessibility. For instance, Mehta et al.^39^ employed macular OCT volumes, color fundus photographs, and demographic data from the UK Biobank—a repository renowned for its standardized, high-quality multimodal datasets—to detect glaucoma. In contrast, Multi-Glau is uniquely designed for real-world clinical constraints. It achieves robust diagnostic accuracy (AUC = 0.8650) even with missing modalities, outperforming state-of-the-art missing-data methods like MMD^30^ (AUC = 0.8485) and Cheerla et al.^31^. (AUC = 0.8592). This breakthrough bridges the gap between idealized “complete-data” research frameworks and the realities of under-resourced clinics, where diagnostic tests are often unavailable. By enabling reliable diagnosis in secondary hospitals lacking advanced equipment or specialized technicians, the system directly addresses critical care shortages—from rural China to underserved communities globally.

Multi-Glau introduces a four-stage glaucoma severity classification framework (early, moderate, advanced, severe) to support precise clinical decision-making, providing guidance on management options—including monitoring, pharmacotherapy, and surgical evaluation—while maintaining adaptability to individual patient profiles^41,42^. Compared to (1) visual field classification models proposed by Xue et al.^43^ and Huang et al.^44^, which collapsed four-stage systems into three tiers by manually setting a fixed IOP threshold (21 mmHg), and (2) unsupervised models^45^ for visual field damage developed without clinical guidance and often lacking interpretability, our M^3^-VF module achieves direct alignment between algorithmic outputs and clinical staging without manual intervention, by integrating multimodal data including OCT, VF, and clinical parameters. Multi-Glau achieved an AUC of 0.9516 and surpassing state-of-the-art models such as DAFT^32^ (AUC = 0.8988) and HoFN^33^ (AUC = 0.9092), demonstrating the precise diagnosis potential of Multi-Glau. When physicians assessed the severity of VF damage using only clinical data and OCT scans—without reviewing the actual VF results—the Multi-Glau system significantly boosted their performance. The greatest improvement was seen when physicians adhered to the model’s recommendations for severe glaucoma cases. This capability stems from its sensitivity to subtle OCT biomarkers (e.g., retinal nerve fiber layer thinning) that human observers often miss in advanced “floor effect” cases^46^. Crucially, Multi-Glau bridges a critical gap in resource-limited settings: by enabling precise VF staging without mandatory VF testing, it empowers clinics lacking advanced perimetry to deliver guideline-aligned care.

Our XGBoost-based glaucoma screening module introduces a paradigm shift by eliminating reliance on imaging data. Instead, it utilizes simple and accessible clinical parameters—age, gender, IOP, CDR, and BCVA—which are routinely collected in basic clinical settings. This design directly addresses the infrastructure limitations of China’s primary care system^1^. In contrast, existing AI-driven screening models, such as that of Oh et al.^47^, depend on high-resolution fundus photographs and RNFL thickness measurements, which require advanced imaging equipment and trained personnel. Our model, by comparison, is well-suited to rural healthcare infrastructure: elderly patients in remote areas can be screened via basic telemedicine or even phone consultations, during which clinicians gather demographic information, symptom reports, and IOP/CDR values—without the need for imaging devices or patient travel. Additionally, the Multi-Glau system incurs low deployment costs, as it supports offline inference on Central Processing Unit-only devices. These characteristics enhance its potential for deployment in remote areas without introducing additional burdens. For instance, while Dong et al.’s use of portable smart fundus cameras^48^ addresses the shortage of experienced ophthalmologists, it may increase infrastructure costs in under-resourced settings.

While Multi-Glau represents a significant advance, limitations remain. First, our current study focuses exclusively on glaucoma, whereas many other chronic eye diseases—such as diabetic retinopathy—also face similar healthcare inequities^49^. Future work could explore adapting the framework to address these conditions. Second, incomplete risk factor data (e.g., refractive errors, systemic diseases) due to patient non-compliance necessitates expanded multicenter data collection. Third, although health equity evaluation frameworks exist^50–52^, their integration into our system awaits future collaboration with public health experts. Fourth, validation in diverse global populations is needed to ensure broader applicability, as our current focus on East Asian PACG populations mirrors geographic and diagnostic biases^53^. Finally, we have not employed a modeling-based approach to share knowledge across multi-tier hospitals. In the future, we aim to leverage high-quality data from tertiary hospitals to pretrain feature extractors for lower-tier institutions.

Multi-Glau exemplifies how AI can translate healthcare policies into clinical tools that bridge—rather than widen—resource disparities. By addressing the limitations of prior models (incomplete data handling, and precise glaucoma diagnosis), it provides a scalable blueprint for equitable glaucoma care. This work not only advances ophthalmic AI in China but also offers a replicable framework for low-resource settings worldwide.

## Methods

### Participants and data collection

This retrospective study collected data from the Eye Center of Xiangya Hospital Central South University (Changsha, China), Yiyang Central Hospital (Yiyang, China) and Taojiang County People’s Hospital (Taojiang, China). The study was approved by the hospital’s Institutional Review Board (No. 202309199). Written informed consent was obtained from participants. The study was conducted in accordance with the Declaration of Helsinki.

The multimodal information consisted of four parts. First, text and numerical data were obtained from the electronic medical record system, including types of glaucoma, age, gender, BCVA, IOP, and CDR. It is important to note that we made efforts to collect additional data on glaucoma risk factors, including refractive status and systemic conditions such as hypertension and diabetes. However, among the 3,545 cases with paired data, many elderly patients with visual field defects were unable to cooperate with subjective refraction tests, resulting in incomplete or inaccurate recordings of refractive status. Moreover, since the majority of our patients have angle-closure glaucoma, a condition less frequently associated with systemic risk factors like hypertension and diabetes (which are more common in open angle glaucoma), these factors were excluded from the current version of the system. Second, MD, VFI and PSD values in VF testings were obtained from the Humphrey Field Analyzer (HFA3; Zeiss Meditec, Dublin, CA), which is the same visual field instrument used in three hospitals. Third, RNFL thickness maps were obtained from OCT scans. The peripapillary circular OCT scans in Xiangya Hospital were acquired at a 3.4-mm diameter circle centered on the optic disc (Cirrus 5000; Carl Zeiss Meditec, Dublin, CA). Peripapillary OCT scans in Yiyang datasets (DRI-OCT; Topcon, Tokyo, Japan) and Taojiang datasets (Triton Version; Topcon, Tokyo, Japan) were also collected. Finally, color fundus photographs or fundus images derived from OCT scans. For each eye, VF testing was linked to OCT scans performed within 30 days. We desired to collect these data on the basis that they were widely accepted as valuable information to diagnose glaucoma^19,20^.

The inclusion criteria were: (1) age 18 years or older and (2) complete records of medical history, glaucoma clinical assessment, VF, OCT, and medical history. The exclusion criteria were: (1) patients with coexisting ocular disorders, including high myopia, diabetic retinopathy, hypertensive retinopathy, and ocular tumors. By excluding these cases, we avoided confounding structural changes and preserved the clarity and reliability of our grading system. For instance, high myopia induces anatomical changes in the optic nerve head that can obscure glaucoma-specific features, such as neuroretinal rim thinning and RNFL alterations; (2) patients with secondary glaucoma, such as traumatic glaucoma or neovascular glaucoma. For example, traumatic glaucoma may involve blood in the anterior chamber, which significantly affects IOP and obstructs the light path, complicating the assessment of glaucoma-related visual field damage; (3) patients with VF testing results showing fixation losses of greater than 2/13 or false-positive rates higher than 15%; and (4) OCT images with signal strength lower than 6 for Cirrus OCT, or those affected by motion artifacts or segmentation errors.

### Ground truth labeling

The criteria for ground truth labeling were as follows: For the screening module, glaucoma cases were annotated by reviewing the EMR system with ICD-10 codes starting with H40, and these annotations were verified by glaucoma specialists. The labeling of glaucomatous visual field (VF) defect severity in the Freeze-Missing and M^3^-VF modules was based on the mean deviation (MD) values from VF testing. In the M^3^-VF module, VF severity was categorized into four groups according to the MD value: early (MD ≥ −6 dB), moderate (−6 to −12 dB), advanced (−12 to −20 dB), and severe (MD ≤ −20 dB), adapting the Hodapp-Anderson-Parrish glaucoma staging system^23,24^. In the Freeze-Missing module, glaucoma severity was broadly classified into early and serious (moderate, advanced, and severe) categories.

The ground truth labeling was performed by a consensus of three glaucoma specialists, all of whom are from the Eye Center of Xiangya Hospital, a renowned ophthalmic medical center in central south China. To provide the experts with comprehensive information for accurate labeling, all cases included in the study underwent a full range of glaucoma examinations, complete with definitive diagnoses and visual field results. While some patients lacked electronic images for model training, paper reports were still available, and we were able to retrieve scanned versions of these reports from the EMR system. Two glaucoma specialists (Z.Y., S.W.) labeled the data for the screening, pre-diagnosis, and definitive diagnosis modules. If they were unable to reach an agreement, a senior glaucoma specialist (X.X.) reviewed the discrepancies and made the final decision.

### Data pre-processing

To impute missing data in the original dataset (17 cases missing BCVA, 364 missing CDR, 33 missing IOP), we trained a multiple imputation model using Light Gradient Boosting Machine^54^. The model was applied iteratively, repeating the process five times, and the average of the five imputations was used as the final result.

We applied three preprocessing steps to all images: (1) resampling all images to a resolution of 256×256 using Lanczos interpolation^55^, (2) enhancing the contrast and brightness of fundus images using Contrast Limited Adaptive Histogram Equalization^56^, and (3) applying global normalization (min-max normalization) to scale pixel values to a uniform range of [0, 1]. A polar coordinate transformation was performed on the optic disc area to expand the sampling region during convolution and improve the visibility of subtle features (shown in Supplementary Fig. 5). Interpretative examples of visual field damage classified into four categories were shown in Supplementary Figs. 6 and 7.

**Fig. 7:**
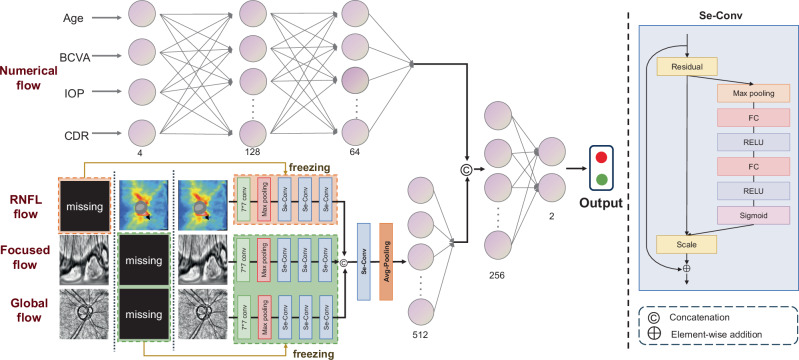
Illustration of the architecture of Freeze-Missing module. The Freeze-Missing module can model missing data for the pre-diagnosis of visual field defects, providing effective support in regions with limited medical resources. The numerical flow extracts high-dimensional features using fully connected layers. The feature extraction networks for RNFL flow, focused flow, and global flow share the same structure, consisting of convolution, max pooling, and Se-Conv layers. Se-Conv can determine the optimal image channels in an end-to-end manner. Image feature fusion is achieved with Se-Conv, and multimodal feature fusion is performed via concatenation. Finally, a fully connected layer is used for class mapping.

### Evaluation metrics

In classification tasks, true positives (*TP*) are instances correctly predicted as positive, and true negatives (*TN*) are instances correctly predicted as negative. False positives (*FP*) are instances incorrectly predicted as positive, and false negatives (*FN*) are instances incorrectly predicted as negative, respectively. Using the definitions above, accuracy, sensitivity, and specificity are calculated as follows:

1 $$\text{Accuracy}=\frac{{TP}+{TN}}{{TP}+{TN}+{FP}+{FN}}$$

2 $${\rm{Sensitivity}}=\frac{{TP}}{{TP}+{FN}}$$

3 $${\rm{Specificity}}=\frac{{TN}}{{TN}+{FP}}.$$

Accuracy indicates the proportion of correctly predicted samples out of the total. Sensitivity reflects the model’s ability to correctly identify positive samples, while specificity reflects its ability to correctly identify negative samples. The area under the receiver operating characteristic curve (AUC) quantifies the overall performance of a model, with values near 1 indicating superior performance.

### Screening module using preliminary clinical data

To develop a screening model suitable for primary care hospitals, we included only five easily accessible features: age, gender, BCVA, IOP, and CDR. We then selected four common models for glaucoma screening: KNN^25^ SVM^26^, LR^27^, and XGB^28^.

KNN predicts based on the majority class among its k nearest neighbors without an explicit learning process. SVM aims to find a hyperplane in the feature space that maximizes the margin between two classes. LR is a linear classification algorithm that models the probability of a binary outcome based on input features. XGB is an ensemble learning algorithm that combines weak learners into strong learners using gradient boosting.

To enhance model performance, we employed Bayesian optimization for hyperparameter tuning across the four models. For instance, we optimized XGB’s maximum depth, learning rate, and number of iterations. Details of the hyperparameter tuning for each model are provided in Supplementary Table 14. Finally, SHAP^29^ was used to conduct interpretability analysis for the screening module.

### Architecture of Freeze-Missing module

A common assumption underlying multimodal learning is the completeness of modalities. However, such an assumption may not always hold in clinical practice since VF testings and OCT scans might not be available in remotely rural secondary hospitals. To address these challenges, we proposed a Freeze-Missing that dealt with missing modalities (Fig. 7).

Figure 7 illustrated the structure of the Freeze-Missing module. The detailed information about convolutional kernels and strides can be found in Supplementary Fig. 8. The model consists of four distinct branches: the global flow processes input fundus images to provide comprehensive global information; the focused flow processes fundus images transformed into polar coordinates to concentrate on the optic disc region; the RNFL flow provides thickness information of the fundus; and the numerical flow incorporates numerical data such as BCVA and IOP.

The concept of “focused flow” contrasts with “global flow”. While “global flow” uses standard fundus images to provide comprehensive information on the disc, retinal nerve fiber layer, and vessels, “focused flow” uses fundus images transformed by polar coordinates. This transformation magnifies the optic cup and disc, improving the extraction of crucial details needed for glaucoma assessment. By shifting from a broad view to a more detailed, localized perspective, this process is termed “focused flow”.

The feature extraction network involved initial feature extraction with a 7 * 7 convolutional kernel, followed by sequential max-pooling and concatenation with three Se-Conv blocks. The Se-Conv embedded the Squeeze-and-Excitation (Se)^57^ block after the Residual^58^, where the Se-block was composed of global pooling, fully connected layers, RELU, and Sigmoid. The Se- block was capable of modeling inter-channel dependencies, allowing for adaptive focusing on more significant channel features. Once feature extraction for global flow, focused flow, and RNFL flow is completed, Freeze-Missing concatenates these features. The merged features then underwent Se-Conv processing to uncover interaction relationships among diverse image sources, focusing on features crucial for visual field grading. Finally, a Multi-Layer Perceptron (MLP) was utilized to blend image and numerical features, ultimately producing visual field damage results.

When image data is missing, Freeze-Missing generates a zero-valued indicator vector of the same dimension for forward propagation and halts parameter updates for the corresponding branch. This ensures that missing data does not interfere with feature extraction from complete modalities. Before feature fusion, the indicator vector is multiplied by a zero vector to ensure that the branch’s feature vector is consistently zero. These steps enable Freeze-Missing to detect the presence of missing data and adjust the parameters accordingly.

### Architecture of M^3^-VF module

We employed fine-grained classification to grade the four subclasses of early, moderate, advanced, and severe visual field severity, so that more precise medical decision could be made. Subtle differences between multiple categories make fine-grained classification challenging, so identifying features with local discriminative power could improve classification performance.

Therefore, the visual field grading model, named M^3^-VF, was created using a multi-perspective, multi-modal, and multi-stage attention mechanism. The input branches of M^3^-VF were consistent with Freeze-Missing-VF, encompassing the global flow, focused flow, RNFL flow, and numerical flow.

**Fig. 8:**
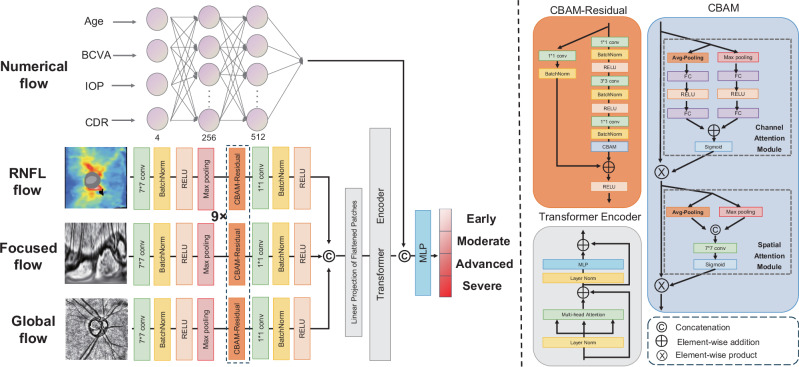
Illustration of the architecture of M^3^-VF module. The M^3^-VF module integrates complete multimodal data to achieve fine-grained, four-class classification of visual field defects, supporting precise diagnostic processes. The numerical flow extracts features using fully connected layers. RNFL flow, focused flow, and global flow share the same structure, composed of convolution, batch normalization, ReLU, max pooling, and CBAM-residual blocks. The CBAM-residual blocks capture important spatial and channel-wise features. Feature fusion is achieved using a transformer encoder, which captures both local and global dependencies between feature vectors. Class mapping is performed through a multilayer perceptron.

Figure 8 illustrated the structure of the M^3^-VF module. The detailed information about convolutional kernels and strides can be found in Supplementary Fig. 9. The feature extraction network shared the same architecture (convolution kernel 7 * 7, Batch Normalization, Max-pooling, nine sequential layers of CBAM-Residual, and convolution kernel 1 * 1). The two-stage attention mechanism comprises the CBAM-Residual for extracting features and the transformer encoder for multimodal integration. The key component of CBAM-Residual is the Convolutional Block Attention Module (CBAM)^59^. CBAM takes the feature map ${\rm{F}}$ as input and then computes the 1- dimensional channel attention map ${{\rm{M}}}_{{\rm{Channel}}}$ and the 2- dimensional spatial attention map ${{\rm{M}}}_{{\rm{Spatial}}}$ sequentially. The overall computation process can be described as follows:

4 $$\begin{array}{c}{{\rm{F}}}^{{\prime} }={{\rm{M}}}_{\mathrm{Channel}}\left({\rm{F}}\right)\otimes {\rm{F}},\\ {{\rm{F}}}^{{\prime} {\prime} }={{\rm{M}}}_{\mathrm{Spatial}}\left({{\rm{F}}}^{{\prime} }\right)\otimes {{\rm{F}}}^{{\prime} }\end{array}$$

where $\otimes$ denotes element-wise multiplication. The numerical flow utilized fully connected layers for non-linear mapping to extract high-order features. After extracting unimodal features, the multi-head attention mechanism in the transformer encoder^60^ is used to capture inter-modal relationships and achieve multimodal information fusion. Multi-head attention originates from self-attention mechanisms, which calculate pairwise similarities between elements:

5 $$\left[q,k,v\right]={\rm{FC}}\left(z\right)$$

6 $$\text{Attention}(\text{q},\text{k},\text{v})={\rm{softmax}}\left(\frac{q{k}^{{\rm{\top }}}}{\sqrt{{D}_{k}}}\right)v$$

The feature map $z$ is subjected to a fully connected mapping to obtain the $q$, $k$, and $v$ vectors. Subsequently, the softmax function is utilized to calculate the attention scores $\text{Attention}(q,{k},{v})$, where the score represents the fusion weights for different modalities. To enhance computational efficiency, parallelizing multiple self-attention operations is known as multi-head attention.

### Comparisons of physicians with and without the assistance of models

To evaluate the computer-aided diagnostic ability of the screening model, Freeze-Missing module, and M^3^-VF module, we further compared the performance of glaucoma physicians with and without the assistance of the three models. The glaucoma physicians (3 experts, 3 senior physicians and 3 junior physicians) were from Xiangya Hospital Central South University. The three experts were professors specializing in glaucoma for over 15 years, the three senior physicians were attendings in glaucoma specialty with over 5 years of practicing experience, and the three junior physicians were residents with 2–4 years of ophthalmology training. None of the physicians have prior information of the testing datasets.

### Statistical analysis and software

Statistical analysis, graph plotting, and primary screening models were carried out using Python 3.8.17, Seaborn 0.12.2 and Scikit-Learn 1.3.0. Freeze-Missing model and M^3^-MF model were built using PyTorch 2.0.1. The accuracy, sensitivity, specificity, AUC were calculated to assess the prediction performance of each model. The clinical benefit and calibration curve are used to evaluate the performance of binary classification models (screening module and pre-diagnostic module). SHAP value was used for determining the feature importance and evaluating interpretability, as elaborated in previous subsections. The parameter settings for the screening task are provided in Supplementary Table 14. The parameters for the pre-diagnosis and definitive diagnosis tasks are listed in Supplementary Table 15.

## Supplementary information

Supplemental material

## Data Availability

The preliminary clinical data and images used in this paper is not publicly available. However, they can be obtained by contacting the first author (zhou_yi@csu.edu.cn) for scientific research purposes.

## Code availability

The source code associated with this study is publicly available at: https://github.com/CowboyH/Multi-Glau.

## References

[1] Li, X. et al. Quality of primary health care in China: challenges and recommendations. *Lancet* **395**, 1802–1812 (2020). 32505251 10.1016/S0140-6736(20)30122-7 PMC7272159

[2] Feng, Z. et al. Long-term care system for older adults in China: policy landscape, challenges, and future prospects. *Lancet* **396**, 1362–1372 (2020). 34338215 10.1016/S0140-6736(20)32136-X

[3] Jayaram, H., Kolko, M., Friedman, D. S. & Gazzard, G. Glaucoma: now and beyond. *Lancet* **402**, 1788–1801 (2023). 37742700 10.1016/S0140-6736(23)01289-8

[4] Sun, Y. et al. Disease burden of glaucoma in China: findings from the global burden of disease 2019 study. *Clin. Epidemiol.* **14**, 827–834 (2022). 35815296 10.2147/CLEP.S357188 PMC9266675

[5] Ittoop, S. M., Jaccard, N., Lanouette, G. & Kahook, M. Y. The role of artificial intelligence in the diagnosis and management of glaucoma. *J. Glaucoma* **31**, 137–146 (2022). 10.1097/IJG.0000000000001972 34930873

[6] Chakravarti, T., Moghimi, S. & Weinreb, R. N. Prediction of central visual field severity in glaucoma. *J. Glaucoma* **31**, 430–437 (2022). 10.1097/IJG.0000000000002031 35649258

[7] Yip, W. et al. 10 years of health-care reform in China: progress and gaps in Universal Health Coverage. *Lancet* **394**, 1192–1204 (2019). 10.1016/S0140-6736(19)32136-1 31571602

[8] The Lancet. A tiered health-care delivery system for China. *Lancet* **393**, 1178. 10.1016/S0140-6736(19)30730-5 30910288

[9] The State Council of the People’s Republic of China. Thirteenth-five year plan for health system reform. *The Central People’s Government of the People’s Republic of China* https://www.gov.cn/zhengce/content/2017-01/09/content_5158053.htm (2017).

[10] The State Council of the People’s Republic of China. Guiding opinions of the General Office of the State Council on promoting the construction of hierarchical diagnosis and treatment system. *The Central People’s Government of the People’s Republic of China* https://www.gov.cn/zhengce/content/2015-09/11/content_10158.htm (2015).

[11] Tang, J. et al. Cost-effectiveness and cost-utility of population-based glaucoma screening in China: a decision-analytic Markov model. *Lancet Glob. Health* **7**, e968–e978 (2019). 10.1016/S2214-109X(19)30201-3 31122906

[12] Uche-Anya, E., Anyane-Yeboa, A., Berzin, T. M., Ghassemi, M. & May, F. P. Artificial intelligence in gastroenterology and hepatology: how to advance clinical practice while ensuring health equity. *Gut* **71**, 1909–1915 (2022). 10.1136/gutjnl-2021-326271 PMC10323754 35688612

[13] Vokinger, K. N., Feuerriegel, S. & Kesselheim, A. S. Mitigating bias in machine learning for medicine. *Commun. Med.* **1**, 25 (2021). 10.1038/s43856-021-00028-w PMC7611652 34522916

[14] Mittermaier, M., Raza, M. M. & Kvedar, J. C. Bias in AI-based models for medical applications: challenges and mitigation strategies. *NPJ Digit. Med.* **6**, 113 (2023). 10.1038/s41746-023-00858-z PMC10264403 37311802

[15] Charilaou, P. & Battat, R. Machine learning models and over-fitting considerations. *World J. Gastroenterol.* **28**, 605–607 (2022). 10.3748/wjg.v28.i5.605 PMC8905023 35316964

[16] Vyas, D. A., Eisenstein, L. G. & Jones, D. S. Hidden in plain sight—reconsidering the use of race correction in clinical algorithms. *N. Engl. J. Med.* **383**, 874–882 (2020). 10.1056/NEJMms2004740 32853499

[17] Chen, T. C. & Mansberger, S. L. Glaucoma Subspecialty Day 2023: Glaucoma Care at the Golden Gate and Beyond. *Am. Acad. Ophthalmol*. https://www.aao.org/Assets/030a5580-872f-463c-98f1-871d8c307c18/638340111998730000/glaucoma-subspecialty-day-2023-syllabus-pdf?inline=1 (2023).

[18] Li, J. O. et al. Digital technology, tele-medicine and artificial intelligence in ophthalmology: a global perspective. *Prog. Retin. Eye. Res.* **82**, 100900 (2021). 10.1016/j.preteyeres.2020.100900 PMC7474840 32898686

[19] Xiong, J. et al. Multimodal machine learning using visual fields and peripapillary circular OCT scans in detection of glaucomatous optic neuropathy. *Ophthalmology* **129**, 171–180 (2022). 10.1016/j.ophtha.2021.07.032 34339778

[20] Li, F. et al. A deep-learning system predicts glaucoma incidence and progression using retinal photographs. *J. Clin. Investig.* **132**, e157968 (2022). 10.1172/JCI157968 PMC9151694 35642636

[21] Pham, Q. T., Han, J. C. & Shin, J. Multimodal deep learning model of predicting future visual field for glaucoma patients. *IEEE Access* **11**, 19049–19058 (2023).

[22] Li, X. et al. The primary health-care system in China. *Lancet* **390**, 2584–2594 (2017). 10.1016/S0140-6736(17)33109-4 29231837

[23] Wang, M. et al. Artificial intelligence classification of central visual field patterns in glaucoma. *Ophthalmology* **127**, 731–738 (2020). 10.1016/j.ophtha.2019.12.004 PMC7246163 32081491

[24] Thompson, A. C., Jammal, A. A., Berchuck, S. I., Mariottoni, E. B. & Medeiros, F. A. Assessment of a segmentation-free deep learning algorithm for diagnosing glaucoma from optical coherence tomography scans. *JAMA Ophthalmol.* **138**, 333–339 (2020). 10.1001/jamaophthalmol.2019.5983 PMC7042899 32053142

[25] Cover, T. & Hart, P. Nearest neighbor pattern classification. *IEEE Trans. Inf. Theory* **13**, 21–27 (1967).

[26] Cortes, C. & Vapnik, V. Support-vector networks. *Mach. Learn* **3**, 273–297 (1995).

[27] DeMaris, A. A tutorial in logistic regression. *J. Marriage Fam.* **4**, 956–968 (1995).

[28] Chen, T. & Guestrin, C. Xgboost: a scalable tree boosting system. *Proc. 22nd ACM SIGKDD International Conference on Knowledge Discovery and Data Mining* 785–794 (Association for Computing Machinery, 2016).

[29] Roth, A. E. *The Shapley Value: Essays in Honor of Lloyd S. Shapley* (Cambridge University Press, 1988).

[30] Cui, Can. et al. Survival prediction of brain cancer with incomplete radiology, pathology, genomic, and demographic data. *International Conference on Medical Image Computing and Computer-Assisted Intervention*, 626–635 (Springer-Verlag, 2022).

[31] Cheerla, A. & Gevaert, O. Deep learning with multimodal representation for pancancer prognosis prediction. *Bioinformatics* **35**, i446–i454 (2019). 10.1093/bioinformatics/btz342 PMC6612862 31510656

[32] Pölsterl, S., Wolf, T. N. & Wachinger, C. Combining 3D image and tabular data via the dynamic affine feature map transform. In *Medical Image Computing and Computer Assisted Intervention* 688–698 (Springer International Publishing, 2021).

[33] Zhou, J. et al. Cohesive multi-modality feature learning and fusion for COVID-19 patient severity prediction. *IEEE Trans. Circuits Syst. Video Technol.* **32**, 2535–2549 (2022). 10.1109/TCSVT.2021.3063952 PMC9280852 35937181

[34] Chattopadhay, A., Sarkar, A., Howlader, P. & Balasubramanian, V. N. Grad-cam++: Generalized gradient-based visual explanations for deep convolutional networks. *IEEE Winter Conference on Applications of Computer Vision (WACV)* 839–847 (Institute of Electrical and Electronics Engineers, 2018).

[35] Springenberg, J. T., Dosovitskiy, A., Brox, T. & Riedmiller, M. A. Striving for simplicity: the all convolutional net. *International Conference on Learning Representations, Workshop Track*. *arXiv preprint arXiv*, 1412.6806 (OpenReview.net, 2014).

[36] Chen, D. et al. Applications of artificial intelligence and deep learning in glaucoma. *Asia Pac. J. Ophthalmol.* **12**, 80–93 (2023). 10.1097/APO.0000000000000596 36706335

[37] Lim, W. S. et al. Use of multimodal dataset in AI for detecting glaucoma based on fundus photographs assessed with OCT: focus group study on high prevalence of myopia. *BMC Med. Imaging* **22**, 206 (2022). 10.1186/s12880-022-00933-z PMC9700928 36434508

[38] Singh, L. K. & Munish, K. A. A novel multimodality based dual fusion integrated approach for efficient and early prediction of glaucoma. *Biomed. Signal Process. Control* **73**, 103468 (2022).

[39] Mehta, P. et al. Automated Detection of Glaucoma With Interpretable Machine Learning Using Clinical Data and Multimodal Retinal Images. *Am. J. Ophthalmol.* **231**, 154–169 (2021). 10.1016/j.ajo.2021.04.021 PMC8560651 33945818

[40] Raghunathan, T., *et al*. Multi-Modal AI/ML Integration for Precision Glaucoma Detection: A Comprehensive Analysis using Optical Coherence Tomography, Fundus Imaging, RNFL, and Vessel Density. In *Proc. 2nd International Conference on Artificial Intelligence and Machine Learning Applications Theme: Healthcare and Internet of Things (AIMLA)* (IEEE, 2024).

[41] Prum, B. E. Jr et al. Primary open-angle glaucoma preferred practice pattern (®) guidelines. *Ophthalmology* **123**, 41–111 (2016). 10.1016/j.ophtha.2015.10.053 26581556

[42] The Advanced Glaucoma Intervention Study (AGIS): 4 comparison of treatment outcomes within race. Seven-year results. Ophthalmol. **105**, 1146–1164 (1998). 10.1016/s0161-6420(98)97013-0 9663215

[43] Xue, Y. et al. A multi-feature deep learning system to enhance glaucoma severity diagnosis with high accuracy and fast speed. *J. Biomed. Inf.* **136**, 104233 (2022). 10.1016/j.jbi.2022.104233 36280089

[44] Huang, X. et al. A structure-related fine-grained deep learning system with diversity data for universal glaucoma visual field grading. *Front. Med.* **9**, 832920 (2022). 10.3389/fmed.2022.832920 PMC8968343 35372429

[45] Yousefi, S. et al. Monitoring glaucomatous functional loss using an artificial intelligence-enabled dashboard. *Ophthalmology* **127**, 1170–1178 (2020). 10.1016/j.ophtha.2020.03.008 PMC7483368 32317176

[46] de Moraes, C. G., Liebmann, J. M., Medeiros, F. A. & Weinreb, R. N. Management of advanced glaucoma: characterization and monitoring. *Surv. Ophthalmol.* **61**, 597–615 (2016). 10.1016/j.survophthal.2016.03.006 27018149

[47] Oh, E., Yoo, T. K. & Hong, S. Artificial neural network approach for differentiating open-angle glaucoma from glaucoma suspect without a visual field test. *Investig. Ophthalmol. Vis. Sci.* **56**, 3957–3966 (2015). 10.1167/iovs.15-16805 26098462

[48] Dong, L. et al. Artificial intelligence for screening of multiple retinal and optic nerve diseases. *JAMA Netw. Open* **5**, e229960 (2022). 10.1001/jamanetworkopen.2022.9960 PMC9066285 35503220

[49] Markle, J. et al. Longitudinal trends and disparities in diabetic retinopathy within an aggregate health care network. *JAMA Ophthalmol.* **142**, 599–606 (2024). 10.1001/jamaophthalmol.2024.0046 PMC11177210 38869883

[50] Liburd, L. C. et al. Addressing health equity in public health practice: frameworks, promising strategies, and measurement considerations. *Annu Rev. Public Health* **41**, 417–432 (2020). 10.1146/annurev-publhealth-040119-094119 31900101

[51] Sun, X. et al. Measurement and analysis of equity in health: a case study conducted in Zhejiang Province, China. *Int J. Equity Health* **17**, 36 (2018). 10.1186/s12939-018-0746-8 PMC5863853 29566758

[52] Wei, H., Jiang, K., Zhao, Y. & Pu, C. Equity of health resource allocation in Chongqing, China, in 2021: a cross-sectional study. *BMJ Open* **14**, e078987 (2024). 10.1136/bmjopen-2023-078987 PMC10806633 38238051

[53] Sun, X. et al. Primary angle closure glaucoma: what we know and what we don’t know. *Prog. Retin Eye Res* **57**, 26–45 (2017). 10.1016/j.preteyeres.2016.12.003 28039061

[54] Ke, G. et al. Lightgbm: a highly efficient gradient boosting decision tree. In *Proc. 31st International Conference on Neural Information Processing Systems* 3149–3157 (Curran Associates, Inc. 2017).

[55] Fadnavis, S. Image interpolation techniques in digital image processing: an overview. *Int. J. Eng. Res. Appl.* **4**, 70–73 (2014).

[56] Pizer, S. M. et al. Contrast-limited adaptive histogram equalization: speed and effectiveness. In *Proc. First Conference on Visualization in Biomedical Computing* 337–345 (Institute of Electrical and Electronics Engineers, 1990).

[57] Hu, J., Shen, L., Albanie, S., Sun, G. & Wu, E. Squeeze-and-excitation networks. *IEEE Trans. Pattern Anal. Mach. Intell.* **8**, 2011–2023 (2020). 10.1109/TPAMI.2019.2913372 31034408

[58] He, K., Zhang, X., Ren, S., Sun, J. Deep residual learning for image recognition. In *Proc. IEEE Conference on Computer Vision and Pattern Recognition* 770–778 (Institute of Electrical and Electronics Engineers, 2016).

[59] Woo, S., Park, J., Lee, J. & Kweon, I. Cbam: convolutional block attention module. In *Proc. European Conference on Computer Vision (ECCV)* 3–19 (Springer, Cham, 2018).

[60] Dosovitskiy, A. et al. An image is worth 16x16 words: transformers for image recognition at scale. In *Proc. International Conference on Learning Representations* 1–21 (OpenReview.net, 2021).

