# Supplementary material for: A three-tier AI solution for equitable glaucoma diagnosis across China’s hierarchical healthcare system
Source: NPJ Digit Med. 2025 Jul 3;8:400. doi: 10.1038/s41746-025-01835-4 (PMC12226717; doi:10.1038/s41746-025-01835-4)
Supplement: Supplementary file 1 — Supplemental material [file 41746_2025_1835_MOESM1_ESM.pdf]

Supplementary Material

Table of Contents

Supplementary Figures

Supplementary Figure 1 Illustration of datasets and ground truth labeling for model construction and external validations.
Supplementary Figure 2 The AUC curve for the optimal model of the Freeze-Missing module under different missing rates.
Supplementary Figure 3 Confusion matrices for the best model of Freeze-Missing.
Supplementary Figure 4 An interpretability study of the Multi-Glau system on optic nerve head changes.
Supplementary Figure 5 Example of CLAHE and Polar Coordinate Transformation.
Supplementary Figure 6 Interpretative examples of visual field damage (VFD) classified into four categories based on fundus images.
Supplementary Figure 7 Interpretative examples of visual field damage (VFD) classified into four categories based on RNFL thickness maps.
Supplementary Figure 8 Structural illustration of the Freeze-Missing module.
Supplementary Figure 9 Structural illustration of the M3-VF module.

Supplementary Tables

Supplementary Table 1 Data characteristics of the healthy and glaucoma cohorts in the Xiangya dataset.
Supplementary Table 2 Data characteristics of the early and serious cohorts in the Xiangya dataset.
Supplementary Table 3 Data characteristics of the early, moderate, advanced, and severe cohorts in the Xiangya dataset.
Supplementary Table 4 Data characteristics of the healthy and glaucoma cohorts in the Taojiang dataset.
Supplementary Table 5 Data characteristics of the early and serious cohorts in the Taojiang dataset.
Supplementary Table 6 Data characteristics of the early, moderate, advanced, and severe cohorts in the Taojiang dataset.
Supplementary Table 7 Data characteristics of the healthy and glaucoma cohorts in the Yiyang dataset.
Supplementary Table 8 Data characteristics of the early and serious cohorts in the Yiyang dataset.
Supplementary Table 9 Data characteristics of the early, moderate, advanced, and severe cohorts in the Yiyang dataset.
Supplementary Table 10 Mean and standard deviation of 5-fold cross-validation.
Supplementary Table 11 Comparison of human-machine interaction accuracy in screening task.
Supplementary Table 12 Comparison of human-machine interaction accuracy in pre-diagnosis task.
Supplementary Table 13 Comparison of human-machine interaction accuracy in definitive diagnosis task.
Supplementary Table 14 Parameters tuning for XGBoost (XGB), Logistic Regression (LR), SVM, and KNN.
Supplementary Table 15 Parameters tuning for the Freeze-Missing and M3-VF module.

Supplementary Figure 1 Illustration of datasets and ground truth labeling for model construction and external validations.

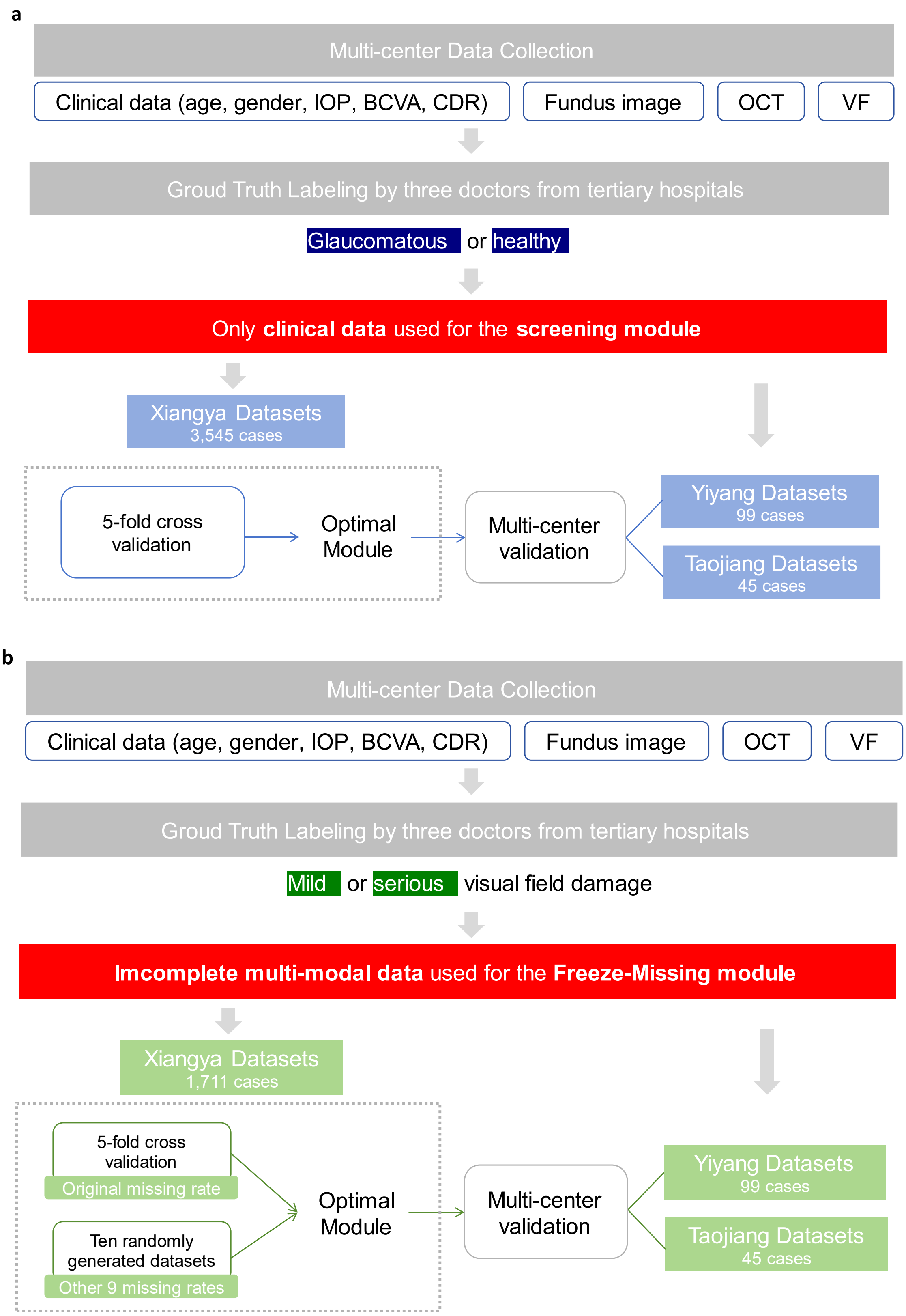

c

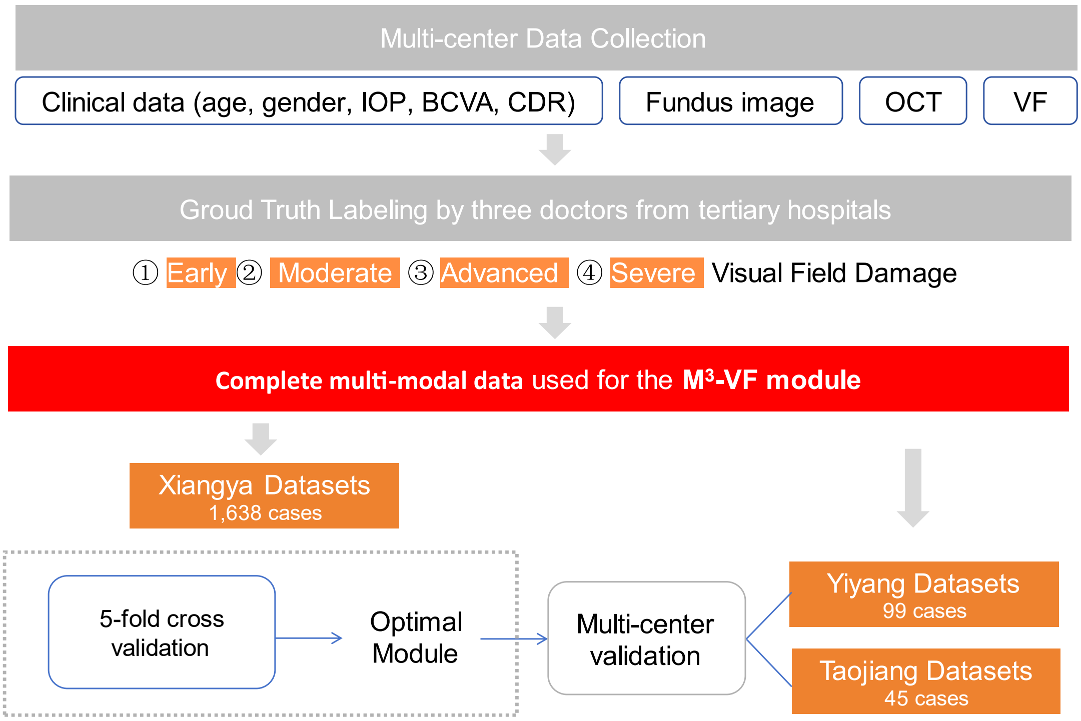

The procedure for data collection and ground truth labeling for the three tasks. **a** Screening module in primary hospitals. **b** Freeze-Missing module for pre-diagnosis in secondary hospitals. **c** M^3-VF module for definitive diagnosis in tertiary hospitals.

Supplementary Figure 2 The AUC curve for the optimal model of the Freeze-Missing module under different missing rates.

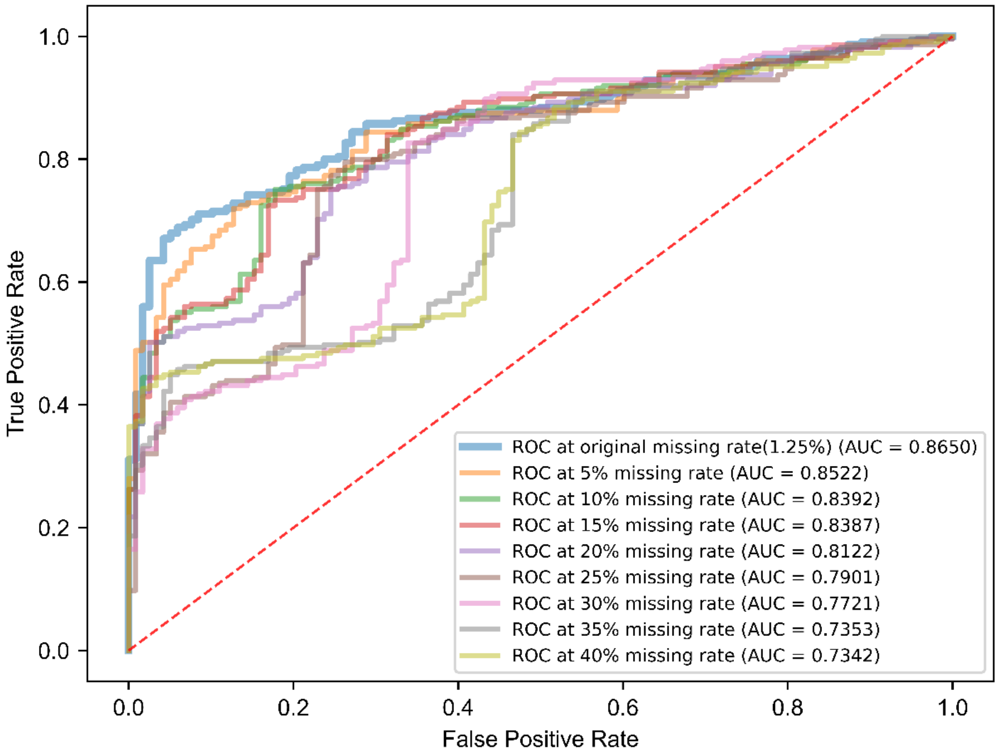

The optimal model during training was determined by accuracy. As the rate of missing data increases, the AUC exhibits a declining trend. The most pronounced changes in the AUC curve occur when the false positive rate ranges from 0.1 to 0.6.

Supplementary Figure 3 Confusion matrices for the best model of Freeze-Missing.

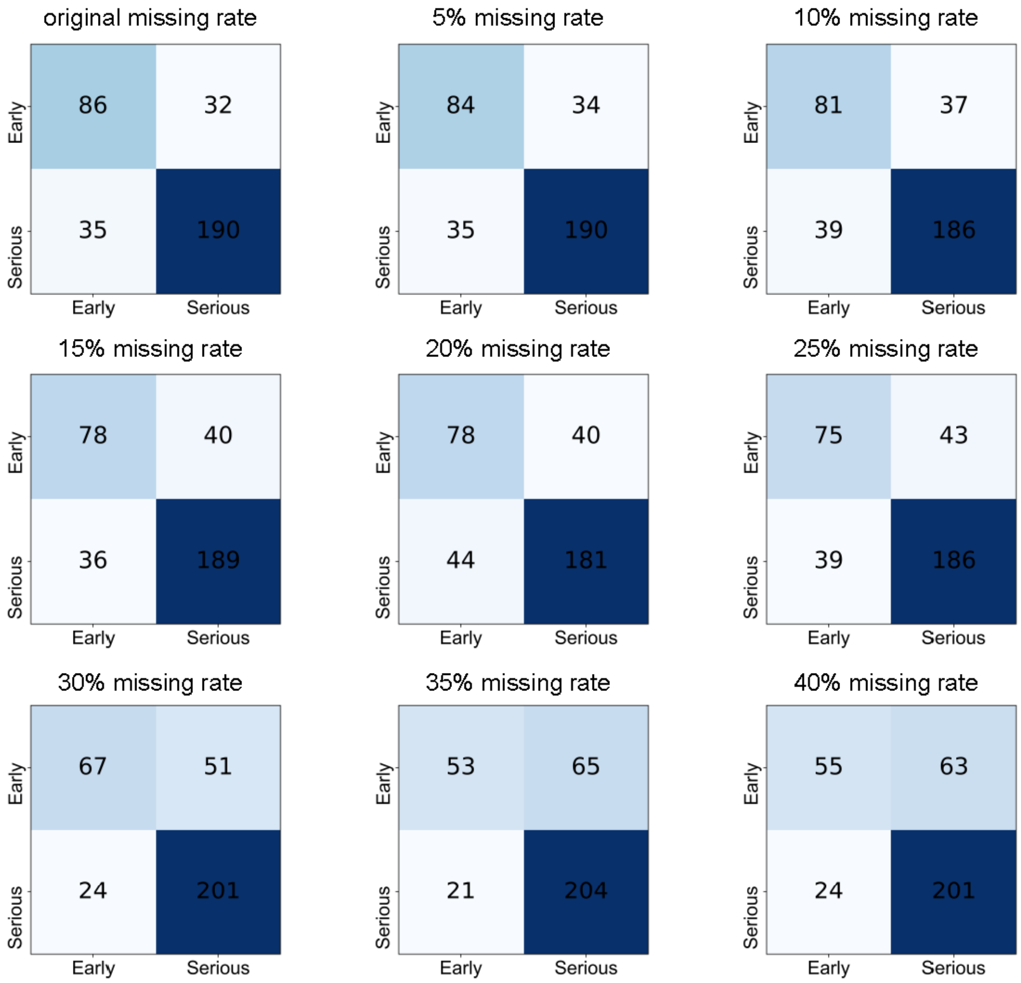

The best module was determined based on the accuracy.

Supplementary Figure 4 An interpretability study of the Multi-Glau system on optic nerve head changes.

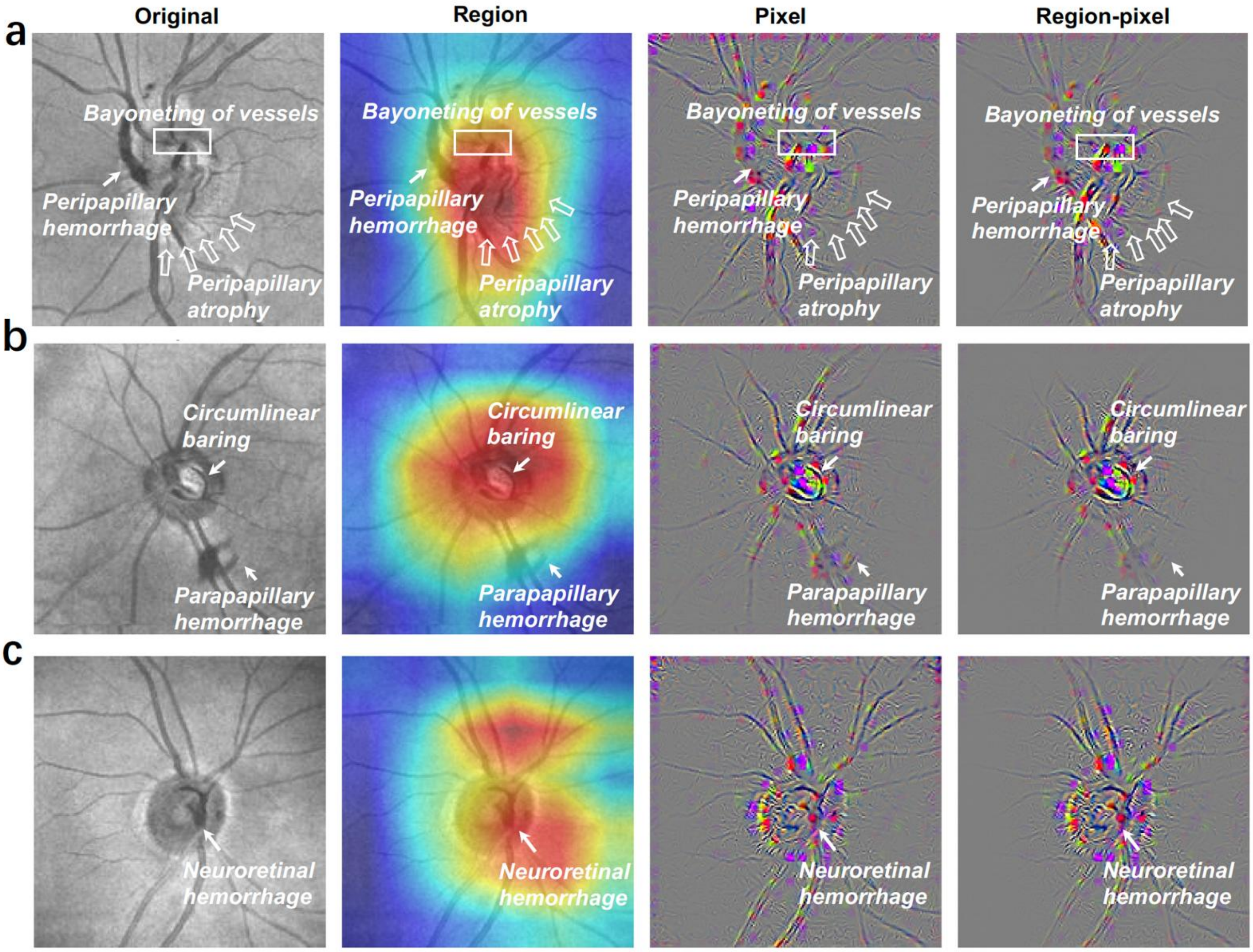

Examples of Multi-Glau's interpretability in detecting disc hemorrhage and other optic nerve head changes, using region-level, pixel-level, and combined region-pixel visualizations. **a** Peripapillary hemorrhage: A significant superior disc hemorrhage emerging from a notch in the superior rim of the disc. This image also highlights peripapillary atrophy (PPA), predominantly temporal to the disc. Additionally, it demonstrates "bayonetting of vessels," where a blood vessel temporarily disappears from view due to significant neuroretinal rim tissue loss, reappearing at the edge of the rim as it navigates along the excavated borders of the optic nerve. **b** Parapapillary hemorrahge: hemorrhage located outside the optic disc accompanied by circumlinear baring of the neuroretinal rim. As the neuroretinal rim tissue is lost, the vessels resting on it may start to follow a circumlinear path around the optic nerve head. **c** Neuroretinal hemorrhage: A hemorrhage located within the neuroretinal rim region.

Supplementary Figure 5 Example of CLAHE and Polar Coordinate Transformation.

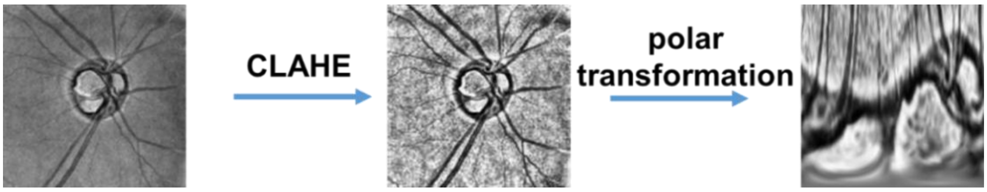

CLAHE is used to enhance the brightness and contrast of the fundus images, and polar coordinate transformation is applied to enlarge the relative size of the optic disc area within the image.

Supplementary Figure 6 Interpretative examples of visual field damage (VFD) classified into four categories based on fundus images.

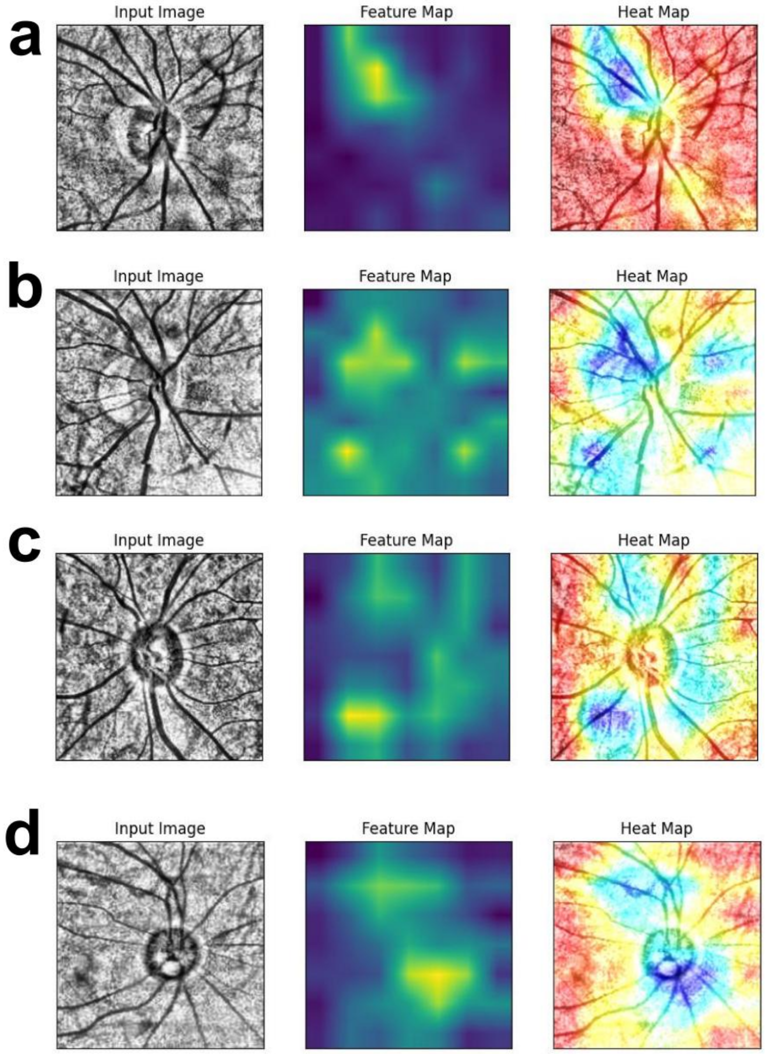

Feature maps and heat maps were generated using the Gradient-weighted Class Activation Mapping (Grad-CAM) technique. **a** Early glaucomatous VFD, **b** moderate glaucomatous VFD, **c** advanced glaucomatous VFD and **d** severe glaucomatous VFD. Left: Original fundus photographs. Middle: Feature maps. Right: Heat maps.

Supplementary Figure 7 Interpretative examples of visual field damage (VFD) classified into four categories based on RNFL thickness maps.

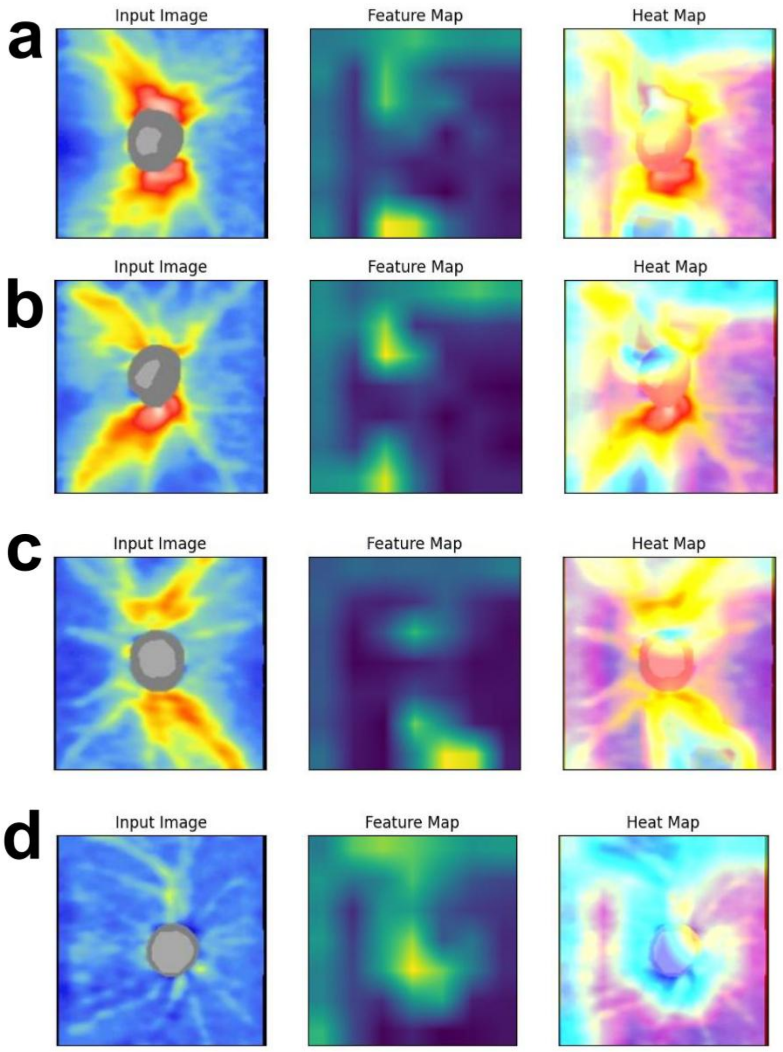

Feature maps and heat maps were generated using the Gradient-weighted Class Activation Mapping (Grad-CAM) technique. **a** Early glaucomatous VFD, **b** moderate glaucomatous VFD, **c** advanced glaucomatous VFD and **d** severe glaucomatous VFD. Left: Original RNFL OCT scans. Middle: Feature maps. Right: Heat maps.

Supplementary Figure 8 Structural illustration of the Freeze-Missing module.

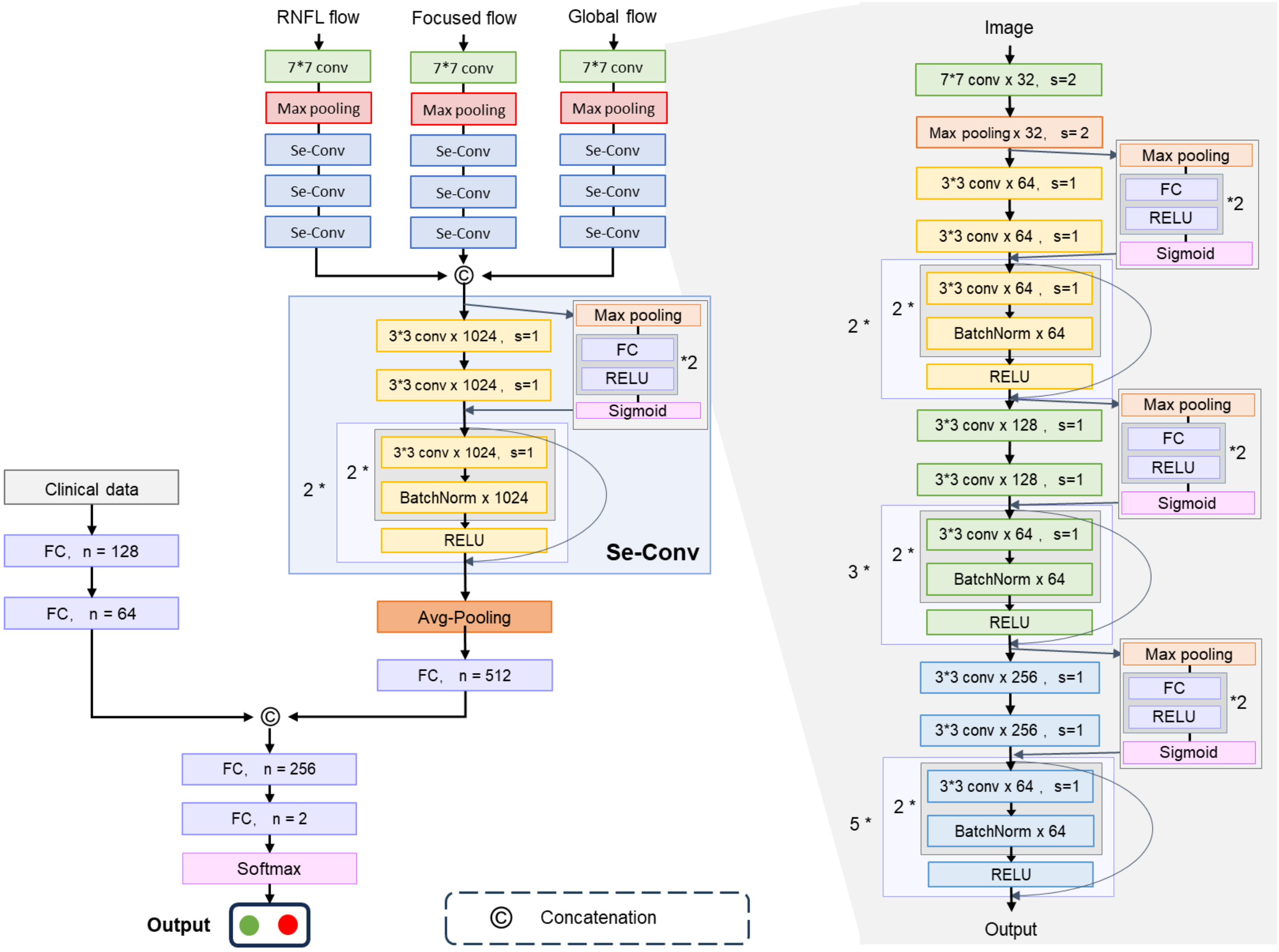

The Freeze-Missing module can model missing data for the pre-diagnosis of visual field defects, providing effective support in regions with limited medical resources. Clinical data are fed into fully connected layers with 128 and 64 neurons, respectively, to extract high-dimensional features. The figure provides detailed parameters for the components used to extract image features, including convolution, pooling, batch normalization, and Se-Conv. The Se-Conv block compresses feature maps using pooling and then employs two fully connected (FC) layers to learn inter-channel correlations, assigning higher weights to channel features more relevant to classification. Convolutional kernels extract features from feature maps. Batch normalization accelerates model convergence and reduces internal covariate shift. ReLU, sigmoid, and softmax are common activation functions used to introduce non-linear transformations. Maximum pooling and average pooling, respectively, take the maximum and average values of the input elements in the pooling window as output.

Supplementary Figure 9 Structural illustration of the M^3-VF module.

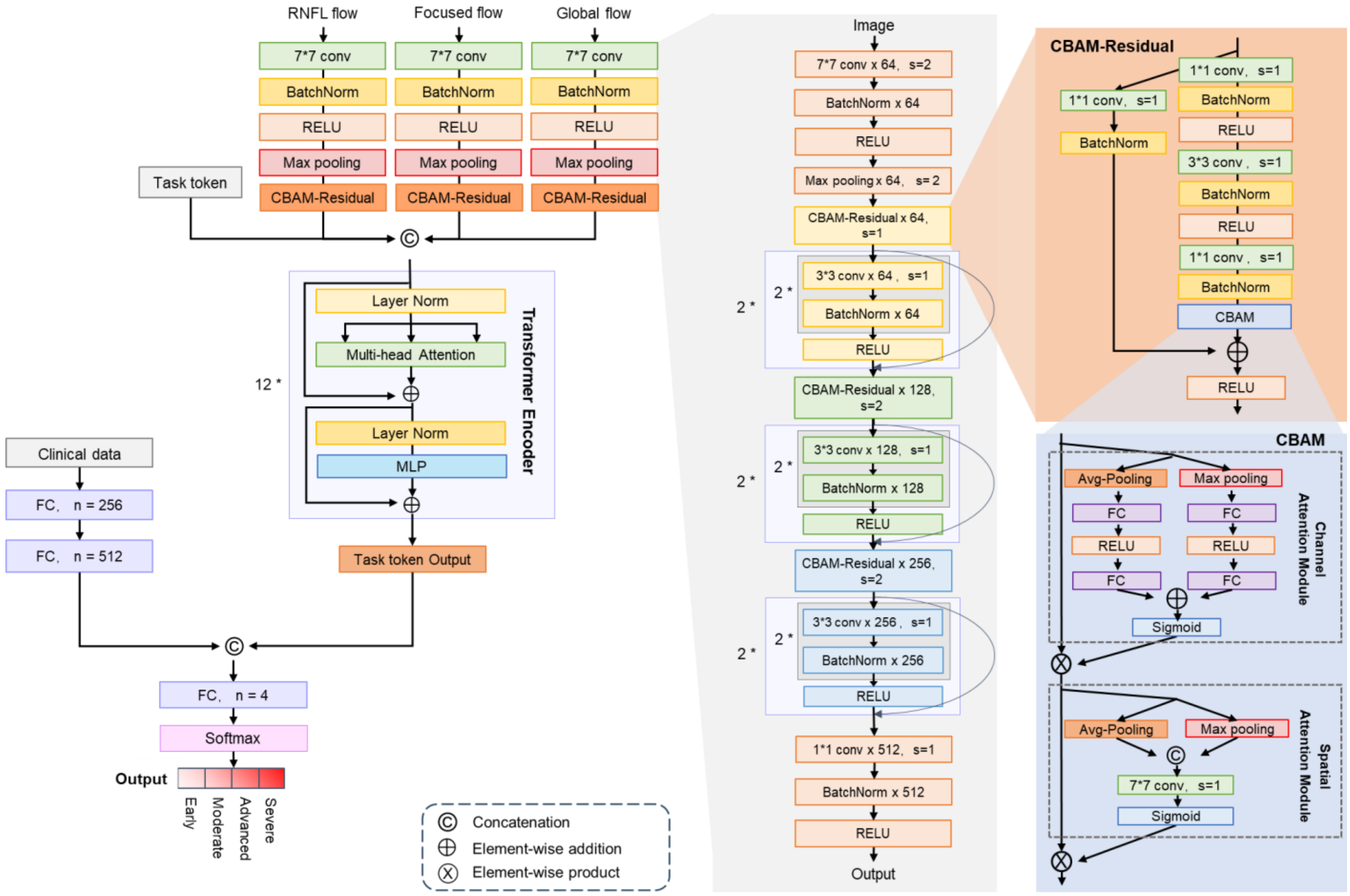

The M^3-VF module integrates complete multimodal data to achieve fine-grained, four-class classification of visual field defects, supporting precise diagnostic processes. Clinical data are processed through two fully connected layers for feature extraction. Features are then extracted using convolution, batch normalization, ReLU, max pooling, and CBAM-Residual. The core component of CBAM-Residual is CBAM, which sequentially infers attention maps along the channel and spatial dimensions, multiplying them with the input feature map for adaptive feature refinement. Feature fusion is achieved using a transformer encoder, where layer normalization ensures stable training and multi-head attention models both local and global dependencies.

	Combined Cohort	G-	G+
**Age**※	60.59±14.11	58.02±12.80	61.18±14.33
**Gender**†			
Male	1682	282	1400
Female	1863	372	1491
**BCVA**※	0.5964±0.4224	0.8907±0.3710	0.5296±0.4045
**CDR**※	0.6504±0.2387	0.4286±0.1337	0.7064±0.2265
**IOP (mmHg)** ※	22.01±11.63	15.73±3.75	23.44±12.33

Supplementary Table 1 Data characteristics of the healthy and glaucoma cohorts in the Xiangya dataset. ※ mean ± standard deviation, †sample size.

	Combined Cohort	Early	Serious
**Age**※	60.72±13.26	58.85±13.10	61.65±13.24
**Gender**†			
Male	804	242	562
Female	907	324	583
**BCVA**※	0.6187±0.4204	0.8766±0.3699	0.4912±0.3840
**CDR**※	0.6636±0.2347	0.5110±0.1705	0.7444±0.2236
**IOP (mmHg)** ※	22.00 ±11.65	18.27 ±7.44	23.87±12.86

Supplementary Table 2 Data characteristics of the early and serious cohorts in the Xiangya dataset. ※ mean ± standard deviation, †sample size.

	Combined Cohort	Early	Moderate	Advanced	Severe
**Age**[※]	60.93 ±12.75	59.34 ±12.23	63.41 ±12.63	62.33 ±11.69	60.56 ±13.50
**Gender**[†]					
Male	765	234	124	109	298
Female	873	319	170	138	246
**BCVA**[※]	0.6283 ±0.4181	0.8728 ±0.3691	0.6051 ±0.3752	0.5555 ±0.3885	0.4252 ±0.3721
**CDR**[※]	0.6625 ±0.2342	0.5109 ±0.1712	0.5811 ±0.2170	0.7146 ±0.2006	0.8402 ±0.1781
**IOP (mmHg)**[※]	21.78 ±11.55	18.18 ±7.41	21.07 ±13.49	23.37 ±11.97	25.16 ±12.55

Supplementary Table 3 Data characteristics of the early, moderate, advanced, and severe cohorts in the Xiangya dataset. [※] mean ± standard deviation, [†]sample size.

	Combined Cohort	G-	G+
**Age**※	64.71±11.38	68.2±2.79	64.28±11.98
**Gender**†			
Male	24	1	23
Female	21	4	17
**BCVA**※	0.2303±0.2543	0.5800±0.2135	0.1866±0.2233
**CDR**※	0.6575±0.2438	0.4000±0.1549	0.6943±0.2317
**IOP (mmHg)** ※	24.18±12.32	17.60±1.85	25.00±12.81

Supplementary Table 4 Data characteristics of the healthy and glaucoma cohorts in the Taojiang dataset. ※ mean ± standard deviation, †sample size.

	Combined Cohort	Early	Serious
**Age**[※]	64.71±11.40	59.56±10.93	66.00±11.15
**Gender**[†]			
Male	24	3	21
Female	21	6	15
**BCVA**[※]	0.2303±0.2543	0.5889±0.2601	0.1407±0.1541
**CDR**[※]	0.6575±0.2438	0.4000±0.2708	0.6306±0.3008
**IOP (mmHg)**[※]	24.18±12.32	19.67±2.98	25.31±13.46

Supplementary Table 5 Data characteristics of the early and serious cohorts in the Taojiang dataset. [※] mean ± standard deviation, [†]sample size.

	Combined Cohort	Early	Moderate	Advanced	Severe
**Age**[※]	64.71 ±11.40	59.56 ±10.93	66.85 ±10.92	64.13 ±13.00	66.27 ±10.10
**Gender**[†]					
Male	24	3	9	3	9
Female	21	6	4	5	6
**BCVA**[※]	0.2303 ±0.2543	0.5889 ±0.2601	0.2046 ±0.1708	0.1381 ±0.1777	0.0867 ±0.0916
**CDR**[※]	0.6575 ±0.2438	0.4000 ±0.2708	0.4923 ±0.1940	0.6000 ±0.2739	0.7667 ±0.3300
**IOP (mmHg)**[※]	24.18 ±12.32	19.67 ±2.98	19.77 ±13.59	24.88 ±12.42	30.33 ±11.84

Supplementary Table 6 Data characteristics of the early, moderate, advanced, and severe cohorts in the Taojiang dataset. [※] mean ± standard deviation, [†]sample size.

	Combined Cohort	G-	G+
**Age**※	64.13±9.54	65.36±7.44	63.98±9.76
**Gender**†			
Male	44	5	39
Female	55	6	49
**BCVA**※	0.4507±0.2843	0.7273±0.1355	0.4161±0.2791
**CDR**※	0.4880±0.2280	0.3364±0.0771	0.5089±0.2339
**IOP (mmHg)** ※	29.92±14.09	16.09±2.54	31.65±13.98

Supplementary Table 7 Data characteristics of the healthy and glaucoma cohorts in the Yiyang dataset. ※ mean ± standard deviation, †sample size.

	Combined Cohort	Early	Serious
**Age**※	64.13±9.54	63.65±10.80	64.27±8.39
**Gender**†			
Male	44	21	23
Female	55	23	32
**BCVA**※	0.4507±0.2843	0.6365±0.2304	0.3020±0.2309
**CDR**※	0.4880±0.2280	0.3320±0.1218	0.5418±0.2939
**IOP (mmHg)** ※	29.92±14.09	27.75±14.16	31.65±13.78

Supplementary Table 8 Data characteristics of the early and serious cohorts in the Yiyang dataset. ※ mean ± standard deviation, †sample size.

	Combined Cohort	Early	Moderate	Advanced	Severe
**Age**[※]	64.13 ±9.54	63.65 ±10.80	66.85 ±10.92	64.44 ±7.64	66.27 ±10.10
**Gender**[†]					
Male	44	21	9	8	6
Female	55	23	18	10	4
**BCVA**[※]	0.4507 ±0.2843	0.6365 ±0.2304	0.4315 ±0.2190	0.2481 ±0.1566	0.0500 ±0.0752
**CDR**[※]	0.4880 ±0.2280	0.3320 ±0.1218	0.3741 ±0.2083	0.5722 ±0.2445	0.9400 ±0.1200
**IOP (mmHg)**[※]	29.92 ±14.09	27.75 ±14.16	27.00 ±12.52	32.72 ±12.41	42.30 ±13.06

Supplementary Table 9 Data characteristics of the early, moderate, advanced, and severe cohorts in the Yiyang dataset. [※] mean ± standard deviation, [†]sample size.

Classifiers	Accuracy	Sensitivity	Specificity	AUC
KNN	0.8725±0.0107	0.8015±0.0209	0.9750±0.0070	0.9694±0.0156
SVM	0.9365±0.0108	0.9419±0.0237	0.9288±0.0128	0.9645±0.0062
LR	0.8325±0.0159	0.8311±0.0260	0.8344±0.0241	0.9024±0.0100
XGB	0.9193±0.0168	0.8961±0.0193	0.9527±0.0207	0.9640±0.0076

Supplementary Table 10 Mean and standard deviation of 5-fold cross-validation.

	Combined cohort			G-			G+		
	**Physi-cian**	**Multi-Glau**	**Physician+ Multi-Glau**	**Physi-cian**	**Multi-Glau**	**Physician+ Multi-Glau**	**Physi-cian**	**Multi-Glau**	**Physician+ Multi-Glau**
**Junior 1**	0.7100	0.8208	0.7700	0.8537	0.8394	0.1667	0.0556	0.7926	0.9024
**Junior 2**	0.8300		0.8700	0.8415		0.7222	0.7778		0.9024
**Junior 3**	0.6900		0.8500	0.6829		0.7222	0.7222		0.8780
**Senior 1**	0.8300		0.8700	0.8780		0.6667	0.6111		0.9146
**Senior 2**	0.6200		0.8700	0.5854		0.7222	0.7778		0.9024
**Senior 3**	0.8500		0.9400	0.8537		0.8889	0.8333		0.9512
**Expert 1**	0.9000		0.9200	0.9756		0.6667	0.5556		0.9756
**Expert 2**	0.8500		0.8800	0.8902		0.8333	0.6667		0.8902
**Expert 3**	0.8600		0.8700	0.8902		0.7778	0.7222		0.8902

Supplementary Table 11 Comparison of human-machine interaction accuracy in screening task.

	Combined cohort			Early cohort			Serious cohort		
	**Physi-cian**	**Multi-Glau**	**Physician+ Multi-Glau**	**Physi-cian**	**Multi-Glau**	**Physician+ Multi-Glau**	**Physi-cian**	**Multi-Glau**	**Physician+ Multi-Glau**
**Junior 1**	0.6800	0.8017	0.8100	0.6866	0.8428	0.7576	0.6667	0.7193	0.8358
**Junior 2**	0.7400		0.7500	0.7313		0.6970	0.7576		0.7761
**Junior 3**	0.6500		0.7800	0.7015		0.6667	0.5455		0.8358
**Senior 1**	0.7000		0.8000	0.7463		0.7273	0.6061		0.8358
**Senior 2**	0.7500		0.7500	0.8358		0.6667	0.5758		0.7910
**Senior 3**	0.7300		0.8000	0.8507		0.6970	0.4848		0.8507
**Expert 1**	0.6600		0.7700	0.6567		0.6364	0.6667		0.8358
**Expert 2**	0.7800		0.7900	0.8806		0.6667	0.5757		0.8507
**Expert 3**	0.7000		0.7600	0.8657		0.6061	0.3636		0.8358

Supplementary Table 12 Comparison of human-machine interaction accuracy in pre-diagnosis task.

		Junior 1	Junior 2	Junior 3	Senior 1	Senior 2	Senior 3	Expert 1	Expert 2	Expert 3
**Combined cohort**	**Physician**	0.5900	0.4500	0.4300	0.5000	0.4500	0.4800	0.4700	0.4600	0.5200
	**Multi- Glau**					0.7547				
	**Physician + Multi-Glau**	0.7500	0.5600	0.5400	0.8200	0.5800	0.6300	0.8100	0.6900	0.6400
**Early cohort**	**Physician**	0.6591	0.5682	0.5682	0.6364	0.6818	0.6818	0.7045	0.6591	0.6136
	**Multi-Glau**					0.7471				
	**Physician + Multi-Glau**	0.8409	0.5909	0.6136	0.8409	0.7500	0.7500	0.8409	0.7955	0.6364
**Moderate cohort**	**Physician**	0.4545	0.4545	0.2727	0.2727	0.0909	0.3636	0.1818	0.1818	0.3636
	**Multi- Glau**					0.6143				
	**Physician + Multi-Glau**	0.8182	0.6364	0.3636	0.9091	0.3636	0.5455	0.5455	0.5455	0.6364
**Advanced cohort**	**Physician**	0.4444	0.1111	0.2222	0.1111	0.2222	0.2222	0.2222	0.4444	0.1111
	**Multi-Glau**					0.6167				
	**Physician + Multi-Glau**	0.4444	0.3333	0.4444	0.7778	0.2222	0.4444	0.7778	0.6667	0.3333
**Severe cohort**	**Physician**	0.5833	0.3889	0.3611	0.5000	0.3333	0.3333	0.3333	0.3056	0.5556
	**Multi-Glau**					0.9245				
	**Physician + Multi-Glau**	0.6944	0.5556	0.5278	0.7778	0.5278	0.5556	0.8611	0.6111	0.7222

Supplementary Table 13 Comparison of human-machine interaction accuracy in definitive diagnosis task.

**XGB**	**Parameter**	max_depth	learning_rate	n_estimators	gamma
	**Value**	(3,8)	(0.01, 0.5)	(50, 500)	(0, 1)
	**Parameter**	subsample	colsample_bytree	reg_alpha	reg_lambda
	**Value**	(0.5, 1)	(0.5, 1)	(0, 1)	(0, 1)
**LR**	**Parameter**	C	penalty	solver	tol
	**Value**	(0.01, 10)	‘none’, ‘l2’, ‘l1’, ‘elasticnet’	‘lbfgs’, ‘liblinear’,’sag’,’saga’	(1e-5, 1e-3)
**SVM**	**Parameter**	C	gamma	kernel	degree
	**Value**	(0.01, 20)	‘scale’, ‘auto’, (0.01, 20)	‘linear’, ‘poly’, ‘rbf’, ‘sigmoid’, ‘precomputed’	(1,5)
**KNN**	**Parameter**	n_neighbors	weights	algorithm	leaf_size
	**Value**	(1, 10)	‘uniform’, ‘distance’	‘auto’,’ball_tree’,’ kd_tree’,’ brute’	(25,35)

Supplementary Table 14 Parameters tuning for XGBoost (XGB), Logistic Regression (LR), SVM, and KNN. Rows labeled "Parameter" indicate the parameters that need adjustment. Rows labeled "Value" denote the range for parameter searching.

	Initial learning rate	Batch size	Optimizer
**Freeze-Missing**	0.0010	16	Adam
Cheerla et al.	0.0002	16	Adam
**MMD**	0.0002	8	Adam
**M^3-VF**	0.0100	20	Adam
**DAFT**	0.0300	20	AdamW
**HoFN**	0.0100	20	Adagrad

Supplementary Table 15 Parameters tuning for the Freeze-Missing and M^3-VF module.
